# Envonalkib Versus Crizotinib in Treatment‐Naive *ALK*‐Positive Non‐Small Cell Lung Cancer: Final Efficacy and Biomarker Analyses of the Randomized Phase III Study

**DOI:** 10.1002/mco2.70998

**Published:** 2026-10-04

**Authors:** Jie Huang, Yuanyuan Wang, Haishuang Sun, Jie Min, Nong Yang, Qitao Yu, Ying Cheng, Yanqiu Zhao, Manxiang Li, Hong Chen, Shouan Ren, Jianying Zhou, Wu Zhuang, Xintian Qin, Lejie Cao, Yan Yu, Jian Zhang, Jianxing He, Jifeng Feng, Hao Yu, Lin Zhang, Xinyuan Zhao, Wenfeng Fang, Jing Li, Li Zhang, Yunpeng Yang

**Affiliations:** ^1^ Department of Medical Oncology State Key Laboratory of Oncology in South China Guangdong Provincial Clinical Research Center for Cancer Sun Yat‐sen University Cancer Center Guangzhou China; ^2^ Department of Oncology The Second Affiliated Hospital of Air Force Medical University Xi'an China; ^3^ Department of Medical Oncology (Lung Cancer and Gastrointestinal Unit) Hunan Cancer Hospital The Affiliated Cancer Hospital of Xiangya School of Medicine Central South University Changsha China; ^4^ Department of Medical Oncology of Respiratory Affiliated Tumor Hospital of Guangxi Medical University Nanning China; ^5^ Department of Thoracic Medical Oncology Jilin Provincial Cancer Hospital Changchun China; ^6^ Department of Medical Oncology The Affiliated Cancer Hospital of Zhengzhou University Zhengzhou China; ^7^ Department of Respiratory First Affiliated Hospital of Xi'an Jiaotong University Xi'an China; ^8^ Department of Respiratory and Critical Care Medicine The First Affiliated Hospital of Chongqing Medical University Chongqing China; ^9^ Department of Respiratory The First Hospital of Shanxi Medical University Taiyuan China; ^10^ Department of Respiratory Disease The First Affiliated Hospital Zhejiang University School of Medicine Hangzhou China; ^11^ Department of Medical Oncology Fujian Cancer Hospital Fuzhou China; ^12^ The First Department of Oncology The First Affiliated Hospital of Guangdong Pharmaceutical University Guangzhou China; ^13^ Department of Respiratory and Critical Care Medicine Anhui Provincial Hospital Hefei China; ^14^ Department of Respiratory Medicine Affiliated Cancer Hospital of Harbin Medical University Harbin China; ^15^ Department of Respiratory Medicine The First Affiliated Hospital of Air Force Medical University Xi'an China; ^16^ Department of Cardiothoracic Surgery The First Affiliated Hospital of Guangzhou Medical College, Guangzhou Research Institute of Respiratory Disease, China State Key Laboratory of Respiratory Disease Guangzhou China; ^17^ Department of Medical Oncology, Jiangsu Cancer Hospital, Jiangsu Institute of Cancer Research The Affiliated Cancer Hospital of Nanjing Medical University Nanjing China; ^18^ Department of Biostatistics Nanjing Medical University Nanjing China; ^19^ Department of Experimental Research State Key Laboratory of Oncology in South China Sun Yat‐sen University Cancer Center Guangzhou China; ^20^ Guangdong Key Laboratory of Nasopharyngeal Carcinoma Diagnosis and Therapy Guangdong Provincial Clinical Research Center for Cancer Guangzhou China

**Keywords:** anaplastic lymphoma kinase, biomarkers, circulating tumor DNA, envonalkib, non‐small cell lung cancer

## Abstract

This study reports efficacy and biomarker results from a multicenter, randomized, open‐label Phase III study (NCT04009317). Treatment‐naive anaplastic lymphoma kinase (*ALK*)‐positive non‐small cell lung cancer (NSCLC) patients were centrally randomized 1:1 to envonalkib or crizotinib, stratified by brain metastases and chemotherapy lines. The primary endpoint was progression‐free survival (PFS) assessed by independent review committee. This analysis updated investigator‐assessed PFS. A total of 264 patients were randomized (envonalkib: 131, crizotinib: 133). Median follow‐up was 46.5 and 46.8 months for envonalkib and crizotinib, respectively. Envonalkib significantly improved PFS over crizotinib (median PFS: 30.4 vs. 12.0 months, *p* < 0.0001; hazard ratio [HR] = 0.48, 95% CI: 0.35–0.65). Overall survival (OS) data remained immature. In the envonalkib group, circulating tumor DNA (ctDNA) analysis revealed poor prognosis for patients with co‐mutations in p53 pathway genes (*TP53*, *CDKN2A*, *MDM2*, *MDM4*) or other concurrent oncogenic drivers (median PFS: 15.7 and 8.3 months). Among 52 patients with post‐progression ctDNA data, putative resistance mechanisms were identified in 14 (26.9%), including secondary *ALK* mutations (*n* = 9) and/or bypass pathway activations (*n* = 9). Final follow‐up confirms the robust benefit of envonalkib over crizotinib, supporting its role as a first‐line option for *ALK*‐positive NSCLC. Biomarker analyses validate the prognostic impact of genomic co‐alterations and delineate resistance mechanisms.

## Graphical abstract

Envonalkib provides robust and durable benefit in treatment‐naïve ALK‐positive NSCLC, with median PFS of 30.4 months versus 12.0 months for crizotinib. Exploratory biomarker analyses showed that concurrent driver alterations and p53 pathway dysfunction reduce treatment efficacy. Both ALK‐dependent and ALK‐independent resistance mechanisms were identified, offering guidance for clinical management.

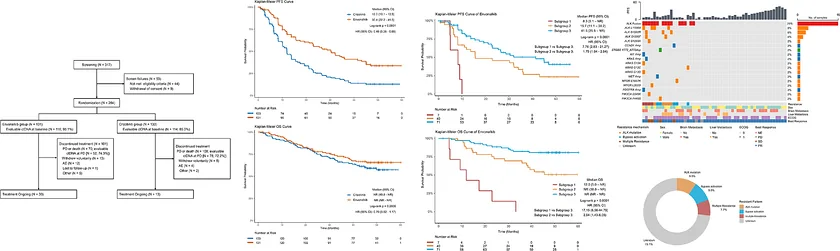

## 1 Introduction

Anaplastic lymphoma kinase (*ALK*) gene fusions are detected in approximately 5% of patients with advanced non‐small cell lung cancer (NSCLC) 10.1038/s41571‐022‐00639‐9 [1], 10.1016/j.lungcan.2017.11.001 [2]. The development of ALK tyrosine kinase inhibitors (TKIs) has significantly improved clinical outcomes in *ALK*‐positive NSCL 10.1056/NEJMoa1408440 [3], 10.1016/j.jtho.2018.06.012 [4], 10.1093/annonc/mdy121 [5], 10.1200/jco.2015.65.5936 [6], 10.1200/jco.2016.71.5904 [7], 10.1016/s2213‐2600(19)30252‐8 [8], 10.1016/s1470‐2045(18)30649‐1 [9]. Envonalkib (TQ‐B3139), a novel second‐generation ALK TKI, has demonstrated superior efficacy and a manageable safety profile compared to crizotinib in the first‐line setting in our randomized Phase III trial (NCT04009317) [10], 10.1038/s41392‐023‐01538‐w [11]. In the primary analysis (data cutoff August 31, 2022), after approximately 28 months of follow‐up, the independent review committee (IRC)‐assessed progression‐free survival (PFS) was significantly improved with envonalkib (median PFS, 24.9 vs. 11.6 months; hazard ratio [HR] = 0.47, 95% confidence interval [CI]: 0.34–0.64, *p* < 0.0001) 10.1038/s41392‐023‐01538‐w [11]. These favorable results led to its approval by the Center for Drug Evaluation in China on June 17, 2024. Despite these promising early results, longer term follow‐up is still needed to fully verify the sustained efficacy of envonalkib.

Recent genomic studies have highlighted the potential impact of concurrent genetic alterations on the efficacy of targeted therapies 10.1016/j.tranon.2022.101471 [12], 10.1093/annonc/mdy333 [13], 10.1016/j.jtho.2023.08.007 [14]. Multiple retrospective studies have suggested that co‐mutations, particularly in the *TP53* gene, are associated with poor prognosis in *ALK*‐positive and other oncogene‐driven NSCLCs 10.1016/j.tranon.2022.101471 [12], 10.1093/annonc/mdy333 [13], 10.1016/j.jtho.2023.08.007 [14], 10.1200/JCO.2020.38.15_suppl.9517 [15], 10.1016/j.jtho.2021.01.1615 [16], 10.1007/s00432‐019‐03065‐0 [17]. Furthermore, evidence suggests that inactivation of *CDKN2A/B* and amplification of *MDM2* may also serve as negative prognostic and predictive markers in *ALK*‐, *EGFR*‐, and *KRAS*‐driven NSCL 10.1016/j.jtho.2023.08.007 [14], 10.1158/2767‐9764.CRC‐24‐0065 [18], 10.1007/s00432‐023‐04924‐7 [19], 10.1038/s43018‐021‐00195‐8 [20], 10.1158/2159‐8290.CD‐22‐1420 [21], 10.1016/j.jtho.2023.05.007 [22], 10.1200/PO.22.00040 [23]. Given that the tumor suppressor p53 is tightly regulated by *MDM2* and *MDM4*, and the p14ARF protein encoded by *CDKN2A*, alterations in these genes are hypothesized to collectively disrupt the p53 pathway and consequently impact prognosis 10.1016/j.ccell.2024.04.009 [24]. However, these findings largely stem from retrospective analyses or small cohorts, underscoring the need for validation in prospective, randomized controlled trials.

In addition to intrinsic genomic factors, acquired resistance to ALK inhibitors also limits response durability. These resistance mechanisms broadly encompass on‐target *ALK* alterations and off‐target bypass pathway activations 10.1158/2159‐8290.Cd‐16‐1123 [25], 10.1002/cac2.12401 [26]. Notably, secondary *ALK* mutations emerge in 50%–60% of patients on second‐generation ALK inhibitors, underscoring the importance of molecular profiling at progression to guide subsequent therapy 10.1158/2159‐8290.Cd‐16‐1123 [25], 10.1158/2159‐8290.Cd‐16‐0596 [27], 10.1016/j.semcancer.2020.07.009 [28], 10.3389/fonc.2022.863461 [29].

Here, we report updated PFS data from the Phase III study of envonalkib versus crizotinib, with a median follow‐up exceeding 46 months (data cutoff February 1, 2024). The present study aimed to characterize the long‐term efficacy of envonalkib versus crizotinib and to define genomic factors associated with treatment outcome and acquired resistance in treatment‐naive *ALK*‐positive NSCLC. With extended follow‐up, envonalkib maintained a substantial PFS advantage over crizotinib. Exploratory analyses of prospectively collected circulating tumor DNA (ctDNA) further identified concurrent oncogenic drivers and p53 pathway dysfunction as adverse prognostic features and delineated both ALK‐dependent and ALK‐independent resistance mechanisms. These findings extend the clinical evidence for envonalkib and highlight the potential value of genomic profiling for risk stratification and treatment selection before therapy and at disease progression.

## 2 Results

### 2.1 Patients and Updated Efficacy

Baseline demographic characteristics of the patients have been reported previously 10.1038/s41392‐023‐01538‐w [11]. A total of 264 patients were randomized to receive either envonalkib or crizotinib (Figure 1). As of the data cutoff on February 1, 2024, with a median follow‐up time of 46.5 months in the envonalkib group and 46.8 months in the crizotinib group, the PFS events reached 53.4% (70/131) in the envonalkib group and 81.2% (108/133) in the crizotinib group. Among censored patients, 22.9% (30/131) and 9.8% (13/133) remained on treatment without progression. The remaining patients discontinued for voluntary withdrawal, adverse events, loss to follow‐up, or other reasons (Figure 1). The updated PFS was consistent with the initial analysis. The investigator (INV)‐assessed PFS analysis confirmed that envonalkib significantly prolonged PFS compared to crizotinib (median PFS: 30.4 vs. 12.0 months, *p* < 0.0001; HR = 0.48, 95% CI: 0.35–0.65) (Figure 2A). Regarding overall survival (OS), events occurred in 32.1% (42/131) and 39.8% (53/133) in the envonalkib group and the crizotinib group, respectively. The median OS was not reached in either group, though a trend of OS benefit was observed with envonalkib (HR = 0.79, 95% CI: 0.52–1.17) (Figure 2B). The updated OS rates at 12, 24, and 36 months were 90.6% (95% CI, 85.7–95.8), 77.7% (95% CI, 70.7–85.3), and 71.8% (95% CI, 64.2–80.2) for the envonalkib group, and 89.4% (95% CI, 84.3–94.8), 76.4% (95% CI, 69.4–84.0), and 65.3% (95% CI, 57.5–74.0) for the crizotinib group, respectively.

**FIGURE 1:**
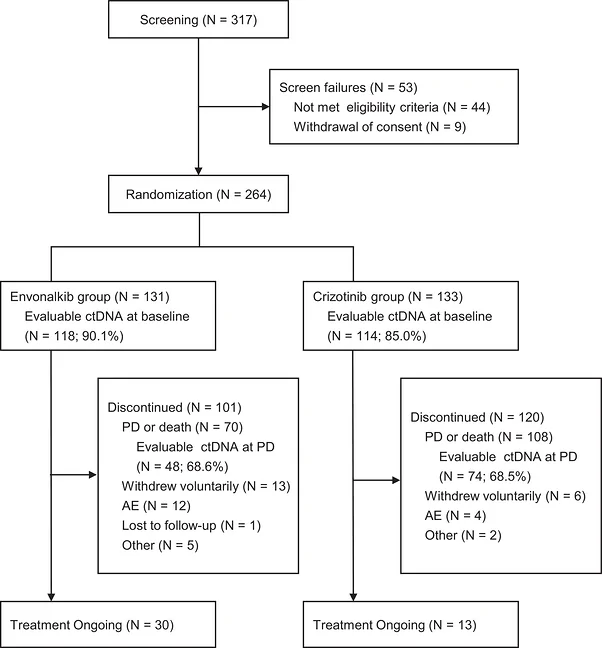
Trial profile. AE, adverse event; ctDNA, circulating tumor DNA; PD, progressive disease.

**FIGURE 2:**
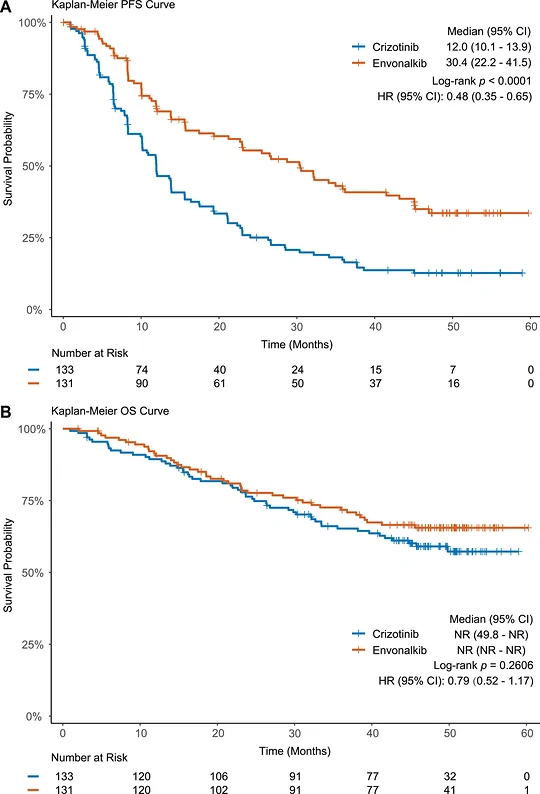
Kaplan–Meier plots for updated investigator‐assessed PFS and OS in the intention‐to‐treat population. (A) Kaplan–Meier curves of PFS by investigator in the two treatment groups. (B) Kaplan–Meier curves of OS in the two treatment groups. CI, confidence interval; HR, hazard ratio; NR, not reached; OS, overall survival; PFS, progression‐free survival.

The impact of baseline brain metastasis status on PFS was assessed through subgroup analyses. Overall, 77 of 264 patients (29.2%) had baseline brain metastases, with 38 (29.0%) in the envonalkib group and 39 (29.3%) in the crizotinib group. Among patients with baseline brain metastases, envonalkib significantly prolonged PFS compared with crizotinib (median PFS: 13.9 months [95% CI: 11.1–30.4] vs. 8.2 months [95% CI: 6.4–11.9], *p* = 0.0011; HR = 0.44, 95% CI: 0.27–0.73). A similar benefit was observed in patients without baseline brain metastases (median PFS: 41.5 months [95% CI: 28.7–NR] vs. 13.9 months [95% CI: 12.0–21.1], *p* < 0.0001; HR = 0.45, 95% CI: 0.30–0.66) (Figure S1).

### 2.2 Prognostic Impact of Baseline ctDNA ALK Fusion

As shown in Figure 1, 232 patients (118 in the envonalkib group; 114 in the crizotinib group) with available baseline plasma sequencing samples were included in the biomarker‐evaluable population (BEP). The mutation landscapes of driver genes, p53 pathway‐related genes, and the most frequently altered genes are presented in Figure S2 (envonalkib group) and Figure S3 (crizotinib group). *ALK* fusion was detected in 72 (61.0%) and 66 (57.9%) patients in the envonalkib and crizotinib groups, respectively. *TP53* was the most frequent concurrent mutation, identified in 40 (33.9%) and 33 (29.2%) patients in the envonalkib and crizotinib groups, respectively.

As shown in Figure S4A, in the envonalkib group, patients with *ALK* fusion detected in ctDNA showed significantly shorter PFS than those without *ALK* fusion detected (median PFS, 17.4 vs. NR months, *p* = 0.0007; HR = 2.56, 95% CI: 1.46–4.48). Although median OS was not reached, patients with *ALK* fusion detected still exhibited a trend toward worse OS (HR = 2.45, 95% CI: 1.16–5.20) (Figure S4B). Similar trends were observed in the crizotinib group (Figure S5A,B). Multivariable Cox regression adjusting for key clinical factors, including tumor burden, brain metastasis, and Eastern Cooperative Oncology Group (ECOG) performance status (PS), confirmed that ctDNA *ALK* fusion status was independently associated with PFS in both the envonalkib arm (HR = 2.28, 95% CI: 1.24–4.21, *p* = 0.008) and the crizotinib arm (HR = 1.61, 95% CI: 1.03–2.51, *p* = 0.037) (Figure S6). In addition, we analyzed the impact of different *ALK* variants on prognosis. The *ALK* variants were detected in 138 patients (72 in the envonalkib group; 66 in the crizotinib group) (Table S1). Variant 3 was the most prevalent variant type (33 [45.8%] in the envonalkib group; 22 [33.3%] in the crizotinib group), followed by Variant 1 (18 [25.0%] in the envonalkib group; 20 [30.3%] in the crizotinib group). No significant differences in PFS or OS were observed among patients with different *ALK* variants in both treatment groups (Figures S7 and S8).

### 2.3 Impact of TP53 Pathway and Driver Mutations

To investigate the prognostic impact of co‐occurring genomic aberrations on patient outcomes, we stratified patients into three molecular subgroups based on alterations detected in ctDNA, beyond the primary *ALK* fusion: the co‐driver group (Subgroup 1), harboring concurrent oncogenic driver alterations other than *ALK* fusions (including *EGFR*, *ERBB2*, *BRAF*, *KRAS*, *MET*, *RET*, *ROS1*, *KIT*, *PIK3CA*, and *MYC*); the p53 Dysfunctional Group (Subgroup 2), lacking concurrent oncogenic drivers but with alterations resulting in p53 dysfunction (*TP53*, *CDKN2A*, *MDM2*, or *MDM4*); and the *ALK*‐Only Group (Subgroup 3), lacking both concurrent oncogenic driver alterations and p53 pathway‐related alterations detected in ctDNA. Importantly, baseline clinical features, including age, sex, ECOG status, tumor burden, and rates of brain metastases, were well‐balanced across these molecular subgroups, mitigating potential confounding (Tables 1 and 2).

**TABLE 1:** Baseline characteristics of participants across molecular subgroups in the biomarker‐evaluable population in the envonalkib cohort.

Characteristic	Subgroup 1 (*N* = 7)	Subgroup 2 (*N* = 40)	Subgroup 3 (*N* = 71)	*p* value
Age (years)				0.733
Mean ± SD	52.4 ± 8.92	55.0 ± 9.52	53.1 ± 11.71	
Median (range)	53 (35,64)	54.5 (37,71)	53.0 (25,75)	
Sex				0.193
Women	1 (14.29)	21 (52.5)	34 (47.89)	
Men	6 (85.71)	19 (47.5)	37 (52.11)	
ECOG status				0.770
0	1 (14.29)	11 (27.5)	16 (22.54)	
1	6 (85.71)	29 (72.5)	55 (77.46)	
Clinical stage				0.405
III	0 (0)	5 (12.5)	4 (5.63)	
IV	7 (100)	35 (87.5)	67 (94.37)	
Tumor burden (mm)				0.122
Mean ± SD	111.2 ± 45.86	79 ± 47.74	72.2 ± 40.54	
Brain metastases				0.887
No	6 (85.71)	29 (72.5)	52 (73.24)	
Yes	1 (14.29)	11 (27.5)	19 (26.76)	
Previous chemotherapy				0.488
No	4 (57.14)	31 (77.5)	50 (70.42)	
Yes	3 (42.86)	9 (22.5)	21 (29.58)	
Smoking status				0.313
Never	3 (42.86)	29 (72.5)	48 (67.61)	
Current/Former	4 (57.14)	11 (27.5)	23 (32.39)	

*Note*: Subgroup 1, patients with concurrent oncogenic driver alterations other than *ALK* fusions detected in ctDNA; Subgroup 2, patients with no concurrent oncogenic drivers detected in ctDNA, but with alterations resulting in p53 dysfunction; Subgroup 3, patients lacking both concurrent oncogenic driver and p53 pathway‐related alterations detected in ctDNA.

Abbreviations: ECOG, Eastern Cooperative Oncology Group; SD, standard deviation.

**TABLE 2:** Baseline characteristics of participants across molecular subgroups in the biomarker‐evaluable population in the crizotinib cohort.

Characteristic	Subgroup 1 (*N* = 7)	Subgroup 2 (*N* = 40)	Subgroup 3 (*N* = 71)	*p* value
Age (years)				0.676
Mean ± SD	55.7 ± 7.53	51.3 ± 11.73	52.3 ± 11.12	
Median (range)	56.5 (43, 63)	53.0 (24, 70)	52.5 (24, 75)	
Sex				0.648
Women	3 (50.00)	13 (38.24)	35 (47.30)	
Men	3 (50.00)	21 (61.76)	39 (52.7)	
ECOG status				0.162
0	0 (0)	10 (29.41)	28 (37.84)	
1	6 (100.00)	24 (70.59)	46 (62.16)	
Clinical stage				0.780
III	0 (0)	3 (8.82)	4 (5.41)	
IV	6 (100.00)	31 (91.18)	70 (94.59)	
Tumor burden (mm)				0.321
Mean ± SD	63.6 ± 27.39	84.4 ± 55.20	63.6 ± 30.37	
Brain metastases				0.577
No	4 (66.67)	27 (79.41)	52 (70.27)	
Yes	2 (33.33)	7 (20.59)	22 (29.73)	
Previous chemotherapy				0.506
No	5 (83.33)	22 (64.71)	56 (75.68)	
Yes	1 (16.67)	12 (35.29)	18 (24.32)	
Smoking status				0.805
Never	3 (50.00)	21 (61.76)	43 (58.11)	
Current/Former	3 (50.00)	13 (38.24)	31 (41.89)	

*Note*: Subgroup 1, patients with concurrent oncogenic driver alterations other than *ALK* fusions detected in ctDNA; Subgroup 2, patients with no concurrent oncogenic drivers detected in ctDNA, but with alterations resulting in p53 dysfunction; Subgroup 3, patients lacking both concurrent oncogenic driver and p53 pathway‐related alterations detected in ctDNA.

Abbreviations: ECOG, Eastern Cooperative Oncology Group; SD, standard deviation.

Next, we analyzed the association between molecular subgroups and clinical outcomes. Compared to Subgroup 3, patients in Subgroups 1 and 2 had significantly worse PFS. The median PFS was 8.3 months (events: 5/7), 15.7 months (events: 26/40), and 41.5 months (events: 32/71) for Subgroups 1, 2, and 3, respectively (*p* < 0.0001). Pairwise comparisons with Benjamini–Hochberg adjustment for multiple testing showed Subgroup 1 versus Subgroup 3, HR = 7.76, 95% CI: 2.83–21.27, adjusted *p* < 0.0001; Subgroup 2 versus Subgroup 3, HR = 1.75, 95% CI: 1.04–2.94, adjusted *p* = 0.034 (Figure 3A). Within Subgroup 2, the median PFS of patients with *TP53* alterations, *CDKN2A* alterations, and *MDM2*/*MDM4* amplification was 15.6, 10.1, and 12.9 months, respectively (Table S2). Although OS data were immature, event rates differed substantially across the groups (Figure 3B): 100% [7/7] for Subgroup 1, 42.5% [17/40] for Subgroup 2, and only 18.3% [13/71] for Subgroup 3. These findings were corroborated in patients receiving crizotinib, where Subgroup 3 also exhibited better PFS and OS compared to Subgroup 1 and Subgroup 2 (Figure S9).

**FIGURE 3:**
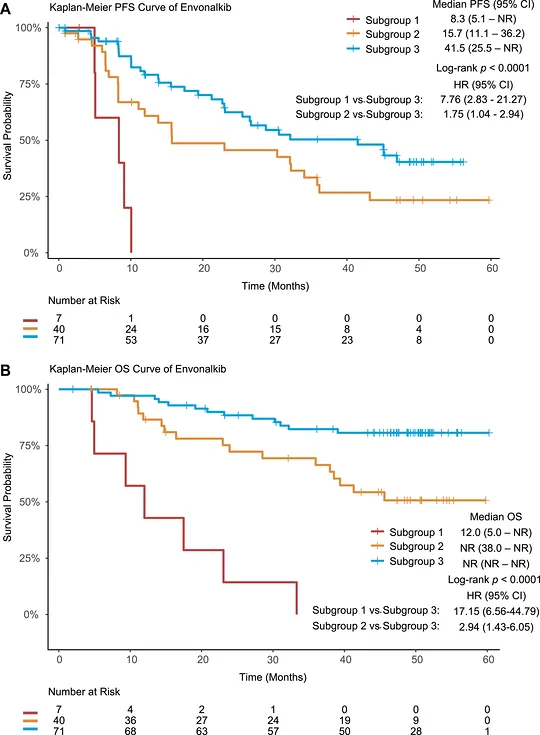
Kaplan–Meier plots for investigator‐assessed PFS and OS in the biomarker‐evaluable population from the envonalkib group. (A) Kaplan–Meier curves of PFS. (B) Kaplan–Meier curves of OS. The patients were divided into three molecular subgroups: Subgroup 1, patients with concurrent oncogenic driver alterations other than *ALK* fusions detected in ctDNA; Subgroup 2, patients with no concurrent oncogenic drivers detected in ctDNA, but with alterations resulting in p53 dysfunction; and Subgroup 3, patients lacking both concurrent oncogenic drivers and p53 pathway‐related alterations detected in ctDNA. *ALK*, anaplastic lymphoma kinase; CI, confidence interval; HR, hazard ratio; NR, not reached; OS, overall survival; PFS, progression‐free survival.

### 2.4 Baseline Features of Long‐Term Responders (LTRs)

Among the 131 patients in the envonalkib cohort, 29 patients (22.1%) who remained progression‐free at data cutoff were identified as LTRs. Conversely, early progressors (EPs) were defined as those progressing within 12 months (*n* = 35, 26.7%). Comparative analysis of the 25 available LTRs and 32 EPs, all of whom had evaluable baseline ctDNA, revealed that LTRs exhibited a significantly lower tumor burden and fewer brain metastases than EPs. Molecular profiling provided further mechanistic insight into this difference: LTRs demonstrated significantly lower *ALK* fusion detection rates, fewer p53 pathway‐related mutations, fewer other driver alterations, and lower blood tumor mutation burden (bTMB) levels compared to EPs (Table 3).

**TABLE 3:** Differences in baseline characteristics between early progressors and long‐term responders in the envonalkib biomarker‐evaluable population.

Characteristic	Early progressors, *n* (%), (*N* = 32)	Long‐term responders, *n* (%), (*N* = 25)	*p* value
Age (years)			0.866
Mean ± SD	54.3 ± 11.12	54.4 ± 12.22	
Median (range)	54 (29, 73)	54 (25, 75)	
Sex			1.000
Women	16 (50.00)	12 (48.00)	
Men	16 (50.00)	13 (52.00)	
ECOG status			0.577
0	11 (34.38)	6 (24.00)	
1	21 (65.63)	19 (76.00)	
Clinical stage			0.325
III	5 (15.63)	1 (4.00)	
IV	27 (84.38)	24 (96.00)	
Pathological type			0.790
Adenocarcinoma	29 (90.63)	24 (96.00)	
Other	3 (9.38)	1 (4.00)	
Tumor burden (mm)			0.002
Mean ± SD	89.7 ± 47.63	53.9 ± 35.87	
Brain metastases			0.037
No	19 (59.38)	22 (88.00)	
Yes	13 (40.63)	3 (12.00)	
Liver metastases			0.186
No	24 (75.00)	23 (92.00)	
Yes	8 (25.00)	2 (8.00)	
Previous chemotherapy			0.728
No	23 (71.88)	16 (64.00)	
Yes	9 (28.13)	9 (36.00)	
Smoking status			0.513
Never	22 (68.75)	20 (80.00)	
Current/Former	10 (31.25)	5 (20.00)	
bTMB			0.010
Mean ± SD	5.0 ± 7.17	1.7 ± 2.55	
*ALK* Fusions			0.001
No	7 (21.88)	17 (68.00)	
Yes	25 (78.13)	8 (32.00)	
Group			0.025
Subgroup 1	5 (15.63)	0 (0.00)	
Subgroup 2	14 (43.75)	7 (28.00)	
Subgroup 3	13 (40.63)	18 (72.00)	

*Note*: Subgroup 1, patients with concurrent oncogenic driver alterations other than *ALK* fusions detected in ctDNA; Subgroup 2, patients with no concurrent oncogenic drivers detected in ctDNA, but with alterations resulting in p53 dysfunction; Subgroup 3, patients lacking both concurrent oncogenic driver and p53 pathway‐related alterations detected in ctDNA.

Abbreviations: *ALK*, anaplastic lymphoma kinase; bTMB, blood tumor mutational burden; ECOG, Eastern Cooperative Oncology Group; SD, standard deviation.

### 2.5 Resistance Mechanisms of Envonalkib

Post‐progression ctDNA analysis was performed on 52 of 70 (74.3%) patients who progressed on envonalkib. All 52 samples were successfully analyzed. The remaining 18 patients were not analyzed due to unavailable plasma samples at progression. Baseline characteristics were comparable between patients with and without post‐progression ctDNA data (Table S3). The landscape of resistance‐associated alterations is illustrated in Figure 4. Putative genetic resistance mechanisms were identified in 14 (26.9%) patients. Secondary *ALK* resistance mutations were the most frequent mechanism, found in 9 (17.3%) patients, involving *L1196M* (*n* = 4, 7.7%), *G1202R* (*n* = 3, 5.8%), *S1206F* (*n* = 2, 3.8%), and *G1269A* (*n* = 1, 1.9%), with one patient (1.9%) harboring a compound mutation (*G1202R* plus *S1206F*). ALK‐independent bypass pathway activation was identified in nine patients (17.3%), involving alterations in genes such as *KRAS* (*n* = 3, 5.8%), *MTOR* (*n* = 2, 3.8%), *PIK3CA* (*n* = 2, 3.8%), *CCND1* (*n* = 1, 1.9%), *ERBB2* (*n* = 1, 1.9%), *KIT* (*n* = 1, 1.9%), *MET* (*n* = 1, 1.9%), and *PDGFRA* (*n* = 1, 1.9%). Notably, four patients (7.7%) harbored both ALK‐dependent and ‐independent resistance mechanisms. Post‐progression ctDNA analysis was also performed on 78 of 108 (72.2%) patients in the crizotinib group, with detailed information provided in Figure S10.

**FIGURE 4:**
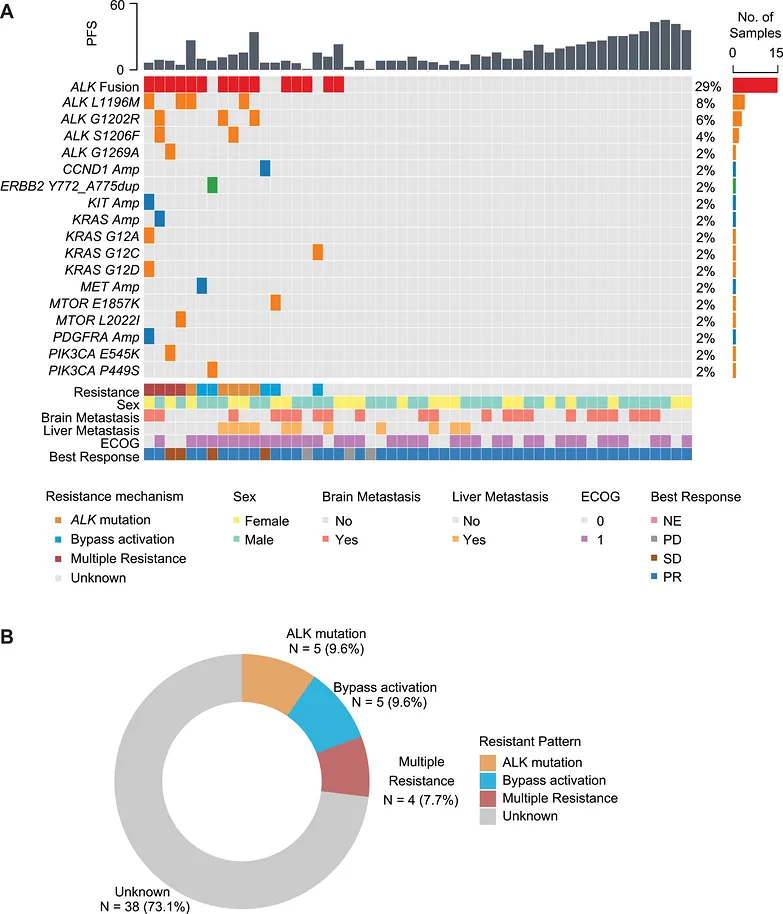
(A) The mutational landscape at the time of disease progression in the envonalkib group. (B) Donut chart summarizing the putative resistance mechanisms. Among the 52 patients with available post‐progression genomic data, resistance mechanisms were categorized as *ALK* mutation only (*n* = 5, 9.6%), bypass activation only (*n* = 5, 9.6%), multiple resistance mechanisms (*n* = 4, 7.7%), and unknown (*n* = 38, 73.1%). *ALK*, anaplastic lymphoma kinase; ECOG, Eastern Cooperative Oncology Group; NE, not evaluable; PD, progressive disease; PR, partial response; SD, stable disease.

## 3 Discussion

The updated PFS data, with a median follow‐up extended to over 46 months, confirm the robust and durable benefit of envonalkib in treatment‐naive *ALK*‐positive NSCLC (mPFS: 30.4 vs. 12.0 months; HR 0.48). These final results are consistent with our preliminary analysis (IRC‐assessed mPFS: 24.9 vs. 11.6 months; HR 0.47), demonstrating the sustained clinical benefit of envonalkib with prolonged follow‐up 10.1038/s41392‐023‐01538‐w [11]. Although crizotinib was the comparator in this study, several second‐generation ALK inhibitors have emerged as standard first‐line therapies in recent years. The median PFS achieved with envonalkib as first‐line treatment is comparable to that of other second‐generation ALK inhibitors. As demonstrated in the ALEX study, alectinib yielded a median PFS of 34.8 months, whereas brigatinib in the ALTA‐1L study showed a median PFS of 30.8 months, and ensartinib in the eXalt3 study reported a median PFS of 25.8 months 10.1016/j.annonc.2020.04.478 [30], 10.1016/j.jtho.2021.07.035 [31], 10.1001/jamaoncol.2021.3523 [32]. Notably, lorlatinib, a third‐generation ALK inhibitor, has shown even longer PFS exceeding 60 months in the CROWN trial, establishing a new standard of care in the first‐line setting10.1200/jco.24.00581 [33]. The availability of multiple ALK inhibitors with different potency and resistance profiles also expands the possibilities for sequential therapy, allowing for individualized treatment strategies from a whole‐course management perspective. Collectively, the comparable efficacy data of envonalkib and other second‐generation ALK inhibitors support its role as a first‐line treatment option for *ALK*‐positive NSCLC. Of note, direct head‐to‐head comparisons between envonalkib and these agents are lacking, and cross‐trial comparisons should be interpreted with caution.

The exploratory biomarker analysis of this study was designed to prospectively validate the prognostic value of concurrent genomic alterations. *ALK*‐positive NSCLC patients derive great clinical benefit from ALK inhibitors, with median OS of approximately 7 years 10.1016/j.annonc.2024.10.662 [34]. However, a few patients still suffer from catastrophic outcomes. The activation in other driver genes has predominantly been reported in patients who developed acquired resistance 10.1016/j.jtho.2019.08.003 [35], 10.1158/1078‐0432.Ccr‐19‐3906 [36], 10.1126/scitranslmed.3003316 [37], 10.1158/1078‐0432.Ccr‐12‐0392 [38], whereas evidence on their intrinsic effects in treatment‐naive *ALK*‐positive NSCLC patients remains limited 10.1093/annonc/mdy333 [13]. In our exploratory biomarker analyses with a relatively large sample size, we are able to investigate the effect of concurrent driver gene alterations at baseline. We found that the prevalence of concurrent driver alterations coexisting with *ALK* fusions in ctDNA before TKI treatment was 5.6% in the total population. Despite the low prevalence, the negative impact of these concurrent drivers was significant: the median PFS was only 8.3 months in patients receiving envonalkib. Besides concurrent drivers, previous studies have also suggested that alterations in p53 pathway‐related genes like *TP53, CDKN2A*, and *MDM2* are associated with poor outcomes in oncogene‐driven NSCLC 10.1016/j.tranon.2022.101471 [12], 10.1093/annonc/mdy333 [13], 10.1016/j.jtho.2023.08.007 [14], 10.1200/JCO.2020.38.15_suppl.9517 [15], 10.1016/j.jtho.2021.01.1615 [16], 10.1007/s00432‐019‐03065‐0 [17], 10.1158/2767‐9764.CRC‐24‐0065 [18]. Our study represents a prospective evaluation of this hypothesis in a randomized cohort. As expected, the prognosis of patients harboring these alterations was suboptimal, with median PFS of 15.7 months in the envonalkib group. Collectively, these findings suggest that patients harboring alterations that lead to bypass activation or p53 dysfunction may require more aggressive treatment strategies other than ALK inhibitor monotherapy.

Using ctDNA from patients with progressive disease, we identified key resistance mechanisms to envonalkib. Secondary *ALK* mutations emerged as the primary mechanism, detected in nine (17%) patients. The most common of these were *L1196M, G1202R*, and *S1206F*, all of which are potentially targetable by other ALK inhibitors. For instance, *L1196M* and *S1206F* can be rescued by brigatinib, ensartinib, and lorlatinib10.1016/j.jtho.2020.11.004 [39], whereas the challenging *G1202R* mutation, a frequent cause of resistance to second‐generation ALK inhibitors, can be effectively addressed by lorlatinib10.1200/jco.18.02236 [40]. Furthermore, we identified several ALK‐independent resistance mechanisms, including *ERBB2* mutation, *KIT* amplification, *KRAS* amplification/mutation, *MET* amplification, *MTOR* mutation, *PDGFRA* amplification, and *PIK3CA* mutation. A notable finding was that a subset of patients (7.7%) harbored both ALK‐dependent and ALK‐independent resistance mechanisms, highlighting the complexity of resistance to targeted therapy. Given this diverse landscape of resistance, our findings strongly support the use of re‐genomic profiling to guide subsequent, personalized treatment strategies for patients who progress on envonalkib.

Certain limitations of our study should be acknowledged. First, this analysis presents INV‐assessed PFS only, as IRC‐assessed PFS was not updated, which may introduce assessment bias in this open‐label trial. In addition, OS data remained immature, and longer follow‐up is needed to confirm whether the PFS benefit translates into an OS advantage. Second, the study was conducted exclusively in a Chinese population, which may limit the generalizability of our findings to other ethnic groups. Third, biomarker analyses had several limitations. Subgroup analyses of specific gene alterations were exploratory and should therefore be interpreted with caution. Additionally, post‐progression ctDNA analysis was feasible in only 74.3% of progressed patients. Despite comparable baseline characteristics, a potential for selection bias cannot be fully excluded. Furthermore, resistance mechanisms may differ between early and late progression, and the current analysis may predominantly reflect the profiles of earlier progression. Fourth, quality of life outcomes were not reported. These limitations should be considered when interpreting our findings.

## 4 Conclusions

Updated results from this study confirm significant clinical improvement with envonalkib in treatment‐naive *ALK*‐positive NSCLC. Exploratory biomarker analyses demonstrated that concurrent driver alterations and p53 dysfunction alterations significantly impaired envonalkib efficacy. This study also revealed ALK‐dependent and ALK‐independent resistance mechanisms to envonalkib, providing guidance for clinical application of envonalkib in patients with *ALK*‐positive NSCLC.

## 5 Materials and Methods

### 5.1 Study Design and Participants

The design of this multicenter, randomized, open‐label, active‐controlled Phase III trial has been previously published 10.1038/s41392‐023‐01538‐w [11]. Key inclusion criteria were (1) age of 18–75 years; (2) ECOG PS score of 0–1; (3) life expectancy ≥12 weeks; (4) histologically or cytologically confirmed locally advanced or metastatic (Stage IIIB–IV) NSCLC with *ALK* fusions; (5) no previous ALK inhibitors as systemic treatment for Stage IIIB–IV NSCLC; (6) <2 lines of chemotherapy for Stage IIIB–IV NSCLC; and (7) ≥1 measurable lesion(s) (other than brain lesions) in the screening period according to RECIST 1.1. Key exclusion criteria were (1) other malignant tumors; (2) allergy to envonalkib or crizotinib components; (3) other anti‐tumor drugs administered within 4 weeks before the first dose; (4) major surgery within 4 weeks before the first dose; (5) curative radiotherapy or minor surgery within 2 weeks before the first dose; (6) acute toxicity from previous treatment not recovered to grade ≤1; (7) active infection; (8) uncontrolled congestive heart failure; (9) widely distributed interstitial fibrosis or interstitial lung disease; (10) symptomatic central nervous system metastases; or (11) any condition preventing oral drug administration. A mandatory baseline brain imaging assessment (magnetic resonance imaging [MRI] or contrast‐enhanced computed tomography [CT]) was required for all participants at screening to determine baseline brain metastasis status.

This study was conducted in accordance with the principles of the Declaration of Helsinki and Good Clinical Practice. The trial protocol was approved by the local ethics review board at each participating site. All patients provided written informed consent.

### 5.2 Procedures

Patients were enrolled by local INVs at participating sites. All eligible patients were randomized 1:1 to receive envonalkib (600 mg twice daily) or crizotinib (250 mg twice daily) utilizing a central randomization system and additionally stratified according to baseline brain metastasis status (yes vs. no) and number of prior chemotherapy lines (0 vs. 1). In case of toxicity during the study, dose adjustment was considered to 500 and 400 mg BID in the envonalkib group and to 200 mg BID and 250 mg QD in the crizotinib group. In the event that a third dose reduction was needed, the treatment was discontinued.

### 5.3 Endpoints and Assessments

The primary endpoint was IRC‐assessed PFS, defined as the time from randomization to the first occurrence of disease progression or death from any cause, whichever occurred first. Secondary endpoints included INV‐assessed PFS and OS.

Tumor assessments were performed every 6 weeks using contrast‐enhanced MRI or CT scans. IRC and INVs evaluated treatment efficacy according to RECIST v1.1. Baseline tumor burden was defined as the sum of the longest diameters of all target lesions at baseline. For the final efficacy analysis presented in this manuscript, an updated IRC assessment was not performed at the data cutoff; therefore, this report focuses on the analysis of updated INV‐assessed PFS, a key secondary endpoint.

### 5.4 Biomarker Analysis

Plasma samples were collected at baseline and at the time of disease progression (within 28 days after the last dose of study drug or prior to initiation of new antineoplastic therapy, whichever occurred first). Cell‐free DNA was extracted from frozen plasma specimens using the QIAamp Circulating Nucleic Acid Kit (Qiagen, Hilden, Germany) and quantified with the Qubit dsDNA HS Assay Kit (Life Technologies). Library preparation was performed using the Accel‐NGS 2S Hyb DNA Library Kit (SWIFT), and library sizing was assessed with the LabChip GX Touch HT (PerkinElmer). Target enrichment was conducted with a custom‐designed 337‐gene pan‐cancer panel (OrigiMed, Shanghai, China) (Table S4). Post‐capture libraries were sequenced on an Illumina NovaSeq 6000 platform (2 × 150 bp paired‐end reads), with an average raw sequencing depth of 15,000× and a minimum effective depth of 2000×. VarDict and VarScan2 were employed for the detection of single‐nucleotide variations (SNVs) and short inserts/deletions (indels), whereas EXCAVATOR was applied for copy number variation (CNV) analysis, and in‐house pipelines were utilized for gene fusion and long indel identification. Variant filtering employed a multi‐step pipeline: (1) common SNPs were excluded using public databases (1000 Genomes, ESP, ExAC/gnomAD); (2) minimum variant allele frequency thresholds were applied: ≥0.1% for hotspot, ≥0.2% for non‐hotspot mutations; (3) minimum supporting reads were required: ≥2 paired reads for SNVs/indels, ≥3 for classical fusions, and ≥5 for non‐classical fusions and long indels. Tumor mutation burden (TMB) was identified as the number of somatic synonymous and non‐synonymous mutations (base substitutions and indels) per megabase of analyzed DNA.

### 5.5 Statistical Analysis

The statistical analysis of this study has been previously published 10.1038/s41392‐023‐01538‐w [11]. Briefly, the Kaplan–Meier method was used to estimate median PFS and OS, with comparisons made using the stratified log‐rank test. The HR and 95% CI were calculated using a Cox proportional hazards regression model. Categorical variables were described by counts and percentages and compared using either the chi‐square test or Fisher's exact test between groups. Continuous variables were reported as means (standard deviations [SD]) or medians (ranges) and were compared using either the independent samples *t*‐test or a nonparametric test.

All statistical tests were two‐sided; a *p* value less than 0.05 was deemed statistically significant. Statistical analyses were conducted using SPSS software (version 26.0, SPSS Institute, IL, USA) and R (version 4.0).

## Author Contributions

**Jie Huang**: formal statistical analysis, biomarker analysis, data interpretation, and preparation of the original draft. **Yuanyuan Wang**: biomarker analysis, data interpretation, and preparation of the original draft. **Haishuang Sun**: biomarker analysis, data interpretation, and preparation of the original draft. **Jie Min**: clinical investigation, participant enrollment, and data acquisition. **Nong Yang**: clinical investigation, participant enrollment, and data acquisition. **Qitao Yu**: clinical investigation, participant enrollment, and data acquisition. **Ying Cheng**: clinical investigation, participant enrollment, and data acquisition. **Yanqiu Zhao**: clinical investigation, participant enrollment, and data acquisition. **Manxiang Li**: clinical investigation, participant enrollment, and data acquisition. **Hong Chen**: clinical investigation, participant enrollment, and data acquisition. **Shouan Ren**: clinical investigation, participant enrollment, and data acquisition. **Jianying Zhou**: clinical investigation, participant enrollment, and data acquisition. **Wu Zhuang**: clinical investigation, participant enrollment, and data acquisition. **Xintian Qin**: clinical investigation, participant enrollment, and data acquisition. **Lejie Cao**: clinical investigation, participant enrollment, and data acquisition. **Yan Yu**: clinical investigation, participant enrollment, and data acquisition. **Jian Zhang**: clinical investigation, participant enrollment, and data acquisition. **Jianxing He**: clinical investigation, participant enrollment, and data acquisition. **Jifeng Feng**: clinical investigation, participant enrollment, and data acquisition. **Hao Yu**: formal statistical analysis. **Lin Zhang**: formal statistical analysis. **Xinyuan Zhao**: formal statistical analysis. **Wenfeng Fang**: conceptualization and study design. **Jing Li**: supervision of the biomarker analyses and critical revision of the manuscript, formal statistical analysis. **Li Zhang**: conceptualization and study design, supervision of the biomarker analyses, and critical revision of the manuscript. **Yunpeng Yang**: conceptualization and study design, supervision of the biomarker analyses, and critical revision of the manuscript. All authors reviewed and edited the manuscript, approved the final version, and agreed to be accountable for the work.

## Funding

This work was supported by the National Natural Science Foundation of China (Grant Nos. 82473203, 82241232, and 82272789), the Guangdong Pearl River Talents Program (2021QN02Y168 to J.L.), and the Young Talents Program of Sun Yat‐sen University Cancer Center (YTP‐SYSUCC‐0040 to J.L.).

## Ethics Statement

This study was done in accordance with the principles of the Declaration of Helsinki and Good Clinical Practice. The trial protocol was approved by the ethics committee of the leading center (approval number: 2019‐XY‐046), and the study was registered with ClinicalTrials.gov (NCT04009317).

## Consent

All patients provided informed consent before study enrollment.

## Conflicts of Interest

Li Zhang has received research support from Akesobio, AstraZeneca, Biokin Pharma, CSPC Pharma, Hengrui Pharma, Kelun Pharma, Novartis, Pierre‐Fabre, Pfizer, Qilu Pharma, and Roche. No other disclosures were reported.

## Supporting information




**Supporting File 1**: mco270998‐sup‐0001‐SuppMat.pdf

## Data Availability

The data that support the findings of this study are available from the corresponding author upon reasonable request.
