# Supplementary material for: Envonalkib Versus Crizotinib in Treatment‐Naive ALK‐Positive Non‐Small Cell Lung Cancer: Final Efficacy and Biomarker Analyses of the Randomized Phase III Study
Source: MedComm (2020). 2026 Oct 4;7(10):e70998. doi: 10.1002/mco2.70998 (PMC13634175; doi:10.1002/mco2.70998)
Supplement: Supplementary file 1 [file MCO2-7-e70998-s001.pdf]

Envonalkib versus Crizotinib in Treatment-Naive *ALK*-Positive Non-Small Cell Lung Cancer: Final Efficacy and Biomarker Analyses of the randomized phase III study

Running Title: Envonalkib in *ALK*+NSCLC: Final Update & Biomarkers

Jie Huang[1#]; Yuanyuan Wang[1#]; Haishuang Sun[1#]; Jie Min[2]; Nong Yang[3]; Qitao Yu[4]; Ying Cheng[5]; Yanqiu Zhao[6]; Manxiang Li[7]; Hong Chen[8]; Shouan Ren[9]; Jianying Zhou[10]; Wu Zhuang[11]; Xintian Qin[12]; Lejie Cao[13]; Yan Yu[14]; Jian Zhang[15]; Jianxing He[16]; Jifeng Feng[17]; Hao Yu[18]; Lin Zhang[1]; Xinyuan Zhao[1]; Wenfeng Fang[1]; Jing Li[19,20*]; Li Zhang[1*]; Yunpeng Yang[1*]

[1]Department of Medical Oncology, State Key Laboratory of Oncology in South China, Guangdong Provincial Clinical Research Center for Cancer, Sun Yat-sen University Cancer Center, Guangzhou 510060, China;

[2]Department of Oncology, The Second Affiliated Hospital of Air Force Medical University, Xi'an 710038, China;

[3]Department of Medical Oncology (Lung Cancer and Gastrointestinal Unit), Hunan Cancer Hospital, The Affiliated Cancer Hospital of Xiangya School of Medicine, Central South University, Changsha 410013, China;

[4]Department of Medical Oncology of Respiratory, Affiliated Tumor Hospital of Guangxi Medical University, Nanning 530021, China;

[5]Department of Thoracic Medical Oncology, Jilin Provincial Cancer Hospital, Changchun 130012, China;

[6]Department of Medical Oncology, The Affiliated Cancer Hospital of Zhengzhou University, Zhengzhou 450003, China;

[7]Department of Respiratory, First Affiliated Hospital of Xi'an Jiaotong University, Xi'an 710061, China;

[8]Department of Respiratory and Critical Care Medicine, The First Affiliated Hospital of Chongqing Medical University, Chongqing 400050, China;

[9]Department of Respiratory, The First Hospital of Shanxi Medical University, Taiyuan 030001, China;

[10]Department of Respiratory Disease, The First Affiliated Hospital, Zhejiang University School of Medicine, Hangzhou 310003, China;

[11]Department of Medical Oncology, Fujian Cancer Hospital, Fuzhou 350014, China;

[12]The First Department of Oncology, The First Affiliated Hospital of Guangdong Pharmaceutical University, Guangzhou 510699, China;

[13]Department of Respiratory and Critical Care Medicine, Anhui Provincial Hospital, Hefei 230000, China;

[14]Department of Respiratory Medicine, Affiliated Cancer Hospital of Harbin Medical University, Harbin 150000, China;

[15]Department of Respiratory Medicine, The First Affiliated Hospital of Air Force Medical University, Xi'an 710032, China;

[16]Department of Cardiothoracic Surgery, The First Affiliated Hospital of Guangzhou Medical College, Guangzhou Research Institute of Respiratory Disease, China State Key Laboratory of Respiratory Disease, Guangzhou 510120, China;

[17]Department of Medical Oncology, Jiangsu Cancer Hospital, The Affiliated Cancer Hospital of Nanjing Medical University, Jiangsu Institute of Cancer Research, Nanjing 210009, China;

[18]Department of Biostatistics, Nanjing Medical University, Nanjing 211166, China;

[19]Department of Experimental Research, State Key Laboratory of Oncology in South China, Sun Yat-sen University Cancer Center, Guangzhou 510060, China.

[20]Guangdong Key Laboratory of Nasopharyngeal Carcinoma Diagnosis and Therapy, Guangdong Provincial Clinical Research Center for Cancer, Guangzhou, 510060, China.

[#]Jie Huang, Yuanyuan Wang and Haishuang Sun contributed equally.

[*]Corresponding Authors

Jing Li, 651 Dongfeng Road East, Yuexiu District, Guangzhou 510060, China. E-mail: lijing3@sysucc.org.cn.

Li Zhang, 651 Dongfeng Road East, Yuexiu District, Guangzhou 510060, China. E-mail: zhangli@sysucc.org.cn;

Yunpeng Yang, 651 Dongfeng Road East, Yuexiu District, Guangzhou 510060, China.

E-mail: yangyp@sysucc.org.cn.

Supplementary Materials

Table S1. Different types of *ALK* fusion identified at baseline

Table S2. Effects of concurrent alterations on progression-free survival.

Table S3. Differences in baseline characteristics between patients with and without post-progression ctDNA in the envonalkib biomarker-evaluable population

Table S4. List of genes in the 337-gene pan-cancer panel

Figure S1. Kaplan-Meier curves of progression-free survival stratified by baseline brain metastasis status.

Figure S2. The mutation landscape at baseline in the envonalkib group.

Figure S3. The mutation landscape at baseline in the crizotinib group.

Figure S4. Kaplan-Meier plots for patients with *ALK* fusions or without *ALK* fusions in the biomarker-evaluable population from envonalkib cohort.

Figure S5. Kaplan-Meier plots for patients with *ALK* fusions or without *ALK* fusions in the biomarker-evaluable population from crizotinib cohort.

Figure S6. Multivariable Cox regression analysis of PFS by ctDNA *ALK* fusion status and clinical factors.

Figure S7. Kaplan-Meier plots for patients with different *ALK* variants in the biomarker-evaluable population from envonalkib cohort.

Figure S8. Kaplan-Meier plots for patients with different *ALK* variants in the biomarker-evaluable population from crizotinib cohort.

Figure S9. Kaplan-Meier plots for progression-free survival and overall survival in the biomarker-evaluable population from crizotinib cohort.

Figure S10. The mutational landscape at the time of disease progression in the crizotinib group.

Table S1. Different types of *ALK* fusion identified at baseline

ALK fusions	Envonalkib (N = 72), n (%)	Crizotinib (N = 66), n (%)
EML4-ALK variant 1	18 (25.0)	20 (30.3)
EML4-ALK variant 2	11 (15.3)	9 (13.6)
EML4-ALK variant 3	33 (45.8)	22 (33.3)
Other types of *ALK* fusion	10 (13.9)	15 (22.7)

Abbreviations: *ALK*, anaplastic lymphoma kinase; *EML4*, echinoderm microtubule-associated protein-like 4.

Table S2. Effects of concurrent alterations on progression-free survival.

Alterations	Envonalkib (N = 118)		Crizotinib (N = 114)	
	n (%)	Median PFS (Months)	n (%)	Median PFS (Months)
TP53 mutations	40 (33.9)	15.6	33 (28.9)	8.3
TP53 wild-type	78 (66.1)	34.5	81 (71.1)	16.4
CDKN2A mutations	8 (6.8)	10.1	5 (4.4)	6.4
CDKN2A wild-type	110 (93.2)	30.6	109 (95.6)	13.6
MDM2/*MDM4* amplification	7 (5.9)	12.9	3 (2.6)	4.7
MDM2/*MDM4* copy-number neutral	111 (94.1)	32.1	111 (97.4)	13.6

Abbreviations: PFS, progression-free survival; *TP53*, tumor protein p53; *CDKN2A*, cyclin-dependent kinase inhibitor 2A; *MDM2*/*MDM4*, mouse double minute 2/4 homolog.

Table S3. Differences in baseline characteristics between patients with and without post-progression ctDNA in the envonalkib biomarker-evaluable population

Characteristic	With post-progression ctDNA (N = 52) n (%)	Without post-progression ctDNA (N = 18) n (%)	*P* value
Age (years)			0.677
Mean ± SD	51.7 ± 9.99	53.6 ± 11.43	
Median (range)	52.0 (29, 72)	52.5 (36, 73)	
Sex			0.062
Women	21 (40.38)	12 (66.66)	
Men	31 (59.62)	6 (33.33)	
ECOG status			0.761
0	15 (28.85)	4 (22.22)	
1	37 (71.15)	14 (77.78)	
Clinical stage			0.327
III	6 (11.54)	0 (0)	
IV	46 (88.46)	18 (100.00)	
Pathological type			1.000
Adenocarcinoma	48 (92.31)	17 (94.44)	
Other	4 (7.69)	1 (5.56)	
Tumor burden (mm)			0.545
Mean ± SD	9.14 ± 4.67	8.23 ± 3.61	
Brain metastases			1.000
No	30 (57.69)	11 (61.11)	
Yes	22 (42.31)	7 (38.89)	
Liver metastases			1.000
No	41 (78.85)	15 (83.33)	
Yes	11 (21.15)	3 (16.67)	
Previous chemotherapy			0.532
No	40 (76.92)	12 (66.67)	
Yes	12 (23.08)	6 (33.33)	
Smoking status			0.554
Never	35 (67.31)	14 (77.78)	
Current/former	17 (32.69)	4 (22.22)	

Abbreviations: ECOG, Eastern Cooperative Oncology Group; SD, standard deviation; *ALK*, anaplastic lymphoma kinase.

Table S4. List of genes in the 337-gene pan-cancer panel

ABL1	*ABL2*	*ACVR1B*	*ACVR2A*
AKT1	*AKT2*	*AKT3*	*ALK*
AMER1	*APC*	*APOBEC3B*	*AR*
ARAF	*ARID1A*	*ARID1B*	*ARID2*
ASXL1	*ATM*	*ATR*	*ATRX*
AXIN1	*AXIN2*	*AXL*	*B2M*
BAP1	*BARD1*	*BCL2*	*BCL2L11*
BCL6	*BCOR*	*BCORL1*	*BCR*
BLM	*BMPR1A*	*BRAF*	*BRCA1*
BRCA2	*BRD4*	*BRIP1*	*CAMTA1*
CARD11	*CASP8*	*CBL*	*CCND1*
CCND2	*CCND3*	*CCNE1*	*CD274*
CD79A	*CD79B*	*CDC73*	*CDH1*
CDK12	*CDK4*	*CDK6*	*CDK8*
CDKN1A	*CDKN1B*	*CDKN2A*	*CDKN2B*
CDKN2C	*CEBPA*	*CFTR*	*CHD2*
CHD4	*CHEK1*	*CHEK2*	*CIC*
COL1A1	*CRBN*	*CREB3L1*	*CREBBP*
CRKL	*CRLF2*	*CSF1R*	*CSK*
CSNK1A1	*CTCF*	*CTNNA1*	*CTNNB1*
CUL3	*CXCR4*	*CYLD*	*CYP2D6*
DAXX	*DDR2*	*DICER1*	*DNMT3A*
DOT1L	*DPYD*	*EGF*	*EGFR*
EP300	*EPHA3*	*EPHA5*	*EPHA7*
EPHB1	*ERBB2*	*ERBB3*	*ERBB4*
ERCC1	*ERRFI1*	*ESR1*	*ETV6*
EZH2	*FAM135B*	*FANCA*	*FANCC*
FANCD2	*FANCE*	*FANCG*	*FANCL*
FANCM	*FAS*	*FAT1*	*FAT3*
FAT4	*FBXW7*	*FGF19*	*FGF3*
FGF4	*FGFR1*	*FGFR2*	*FGFR3*
FGFR4	*FH*	*FLCN*	*FLT1*
FLT3	*FLT4*	*FOXL2*	*FOXP1*
FUBP1	*FUS*	*FYN*	*GATA1*
GATA2	*GATA3*	*GATA4*	*GLI1*
GLI3	*GNA11*	*GNAQ*	*GNAS*
GRIN2A	*H3-3A*	*HDAC9*	*HGF*
HNF1A	*HRAS*	*HSP90AA1*	*IDH1*
IDH2	*IGF1R*	*IKZF1*	*IL7R*
INPP4B	*ITK*	*JAK1*	*JAK2*
JAK3	*JUN*	*KDM5A*	*KDM5C*

KDM6A	*KDR*	*KEAP1*	*KIT*
KMT2A	*KMT2C*	*KMT2D*	*KRAS*
LMO1	*LRP1*	*LRP1B*	*LZTR1*
MAP2K1	*MAP2K2*	*MAP2K4*	*MAP3K1*
MAP3K13	*MCL1*	*MDM2*	*MDM4*
MED12	*MEN1*	*MET*	*MGMT*
MLH1	*MPL*	*MRE11*	*MSH2*
MSH6	*MTOR*	*MUTYH*	*MYC*
MYCL	*MYCN*	*MYD88*	*NBN*
NCOA2	*NCOR1*	*NEK11*	*NF1*
NF2	*NFE2L2*	*NFIB*	*NFKBIA*
NOTCH1	*NOTCH2*	*NOTCH3*	*NOTCH4*
NPM1	*NR4A3*	*NRAS*	*NRG1*
NRG3	*NSD1*	*NSD2*	*NTRK1*
NTRK2	*NTRK3*	*NUP93*	*PALB2*
PARP1	*PARP4*	*PAX5*	*PBRM1*
PDCD1	*PDCD1LG2*	*PDGFRA*	*PDGFRB*
PIK3CA	*PIK3CB*	*PIK3CD*	*PIK3CG*
PIK3R1	*PIK3R2*	*PMS2*	*POLB*
POLD1	*POLE*	*PPP2R1A*	*PRDM1*
PREX2	*PRKACA*	*PRKCI*	*PRKDC*
PRKN	*PRSS1*	*PRSS8*	*PTCH1*
PTEN	*PTPN11*	*QKI*	*RAC1*
RAD50	*RAD51*	*RAD51B*	*RAD51C*
RAD51D	*RAD54L*	*RAF1*	*RANBP2*
RARA	*RB1*	*RBM10*	*RECQL*
RET	*RHOA*	*RICTOR*	*RNF43*
ROCK1	*ROCK2*	*ROS1*	*RPTOR*
RUNX1	*RUNX1T1*	*SDHA*	*SDHB*
SDHC	*SDHD*	*SETBP1*	*SETD2*
SF3B1	*SIK1*	*SLIT2*	*SMAD2*
SMAD3	*SMAD4*	*SMARCA4*	*SMARCB1*
SMO	*SNCAIP*	*SND1*	*SOCS1*
SOX2	*SOX9*	*SPEN*	*SPINK1*
SPOP	*SPTA1*	*SRC*	*SRSF2*
SSX1	*STAG2*	*STAT3*	*STAT4*
STK11	*SUFU*	*SYK*	*TBX3*
TCF7L2	*TENT5C*	*TERT*	*TET1*
TET2	*TFE3*	*TGFBR1*	*TGFBR2*
TOP1	*TOP2A*	*TP53*	*TP63*
TSC1	*TSC2*	*TSHR*	*U2AF1*
VEGFA	*VHL*	*WEE1*	*WEE2*
WT1	*XPO1*	*XRCC2*	*XRCC3*

ZNF750			

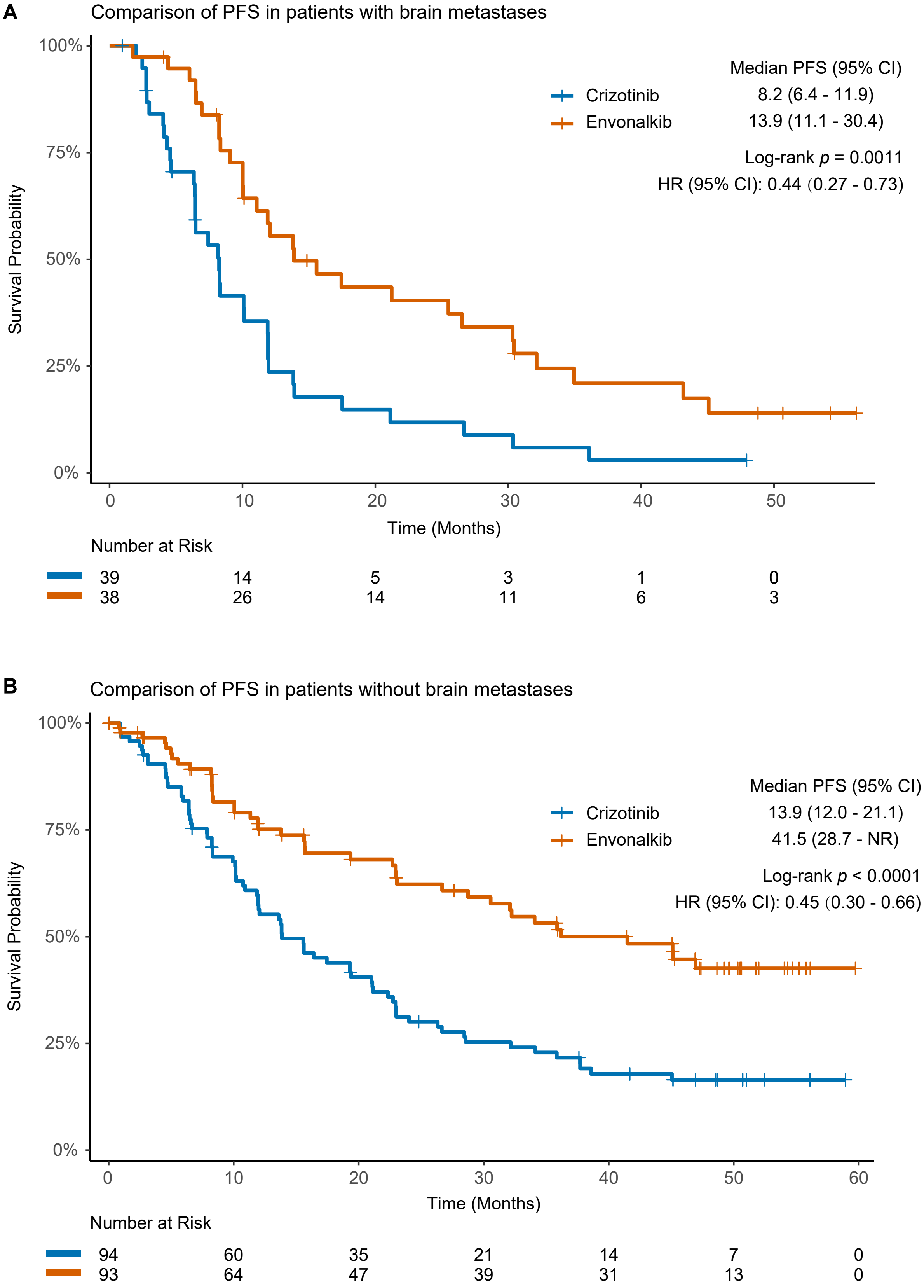

Figure S1. Kaplan-Meier curves of progression-free survival stratified by baseline brain metastasis status.

(A) Kaplan-Meier curves of progression-free survival in patients with baseline brain metastases. (B) Kaplan-Meier curves of progression-free survival in patients without baseline brain metastases. Abbreviations: PFS, progression-free survival; HR, hazard ratio; CI, confidence interval; NR, not reached; No. at risk, number at risk.

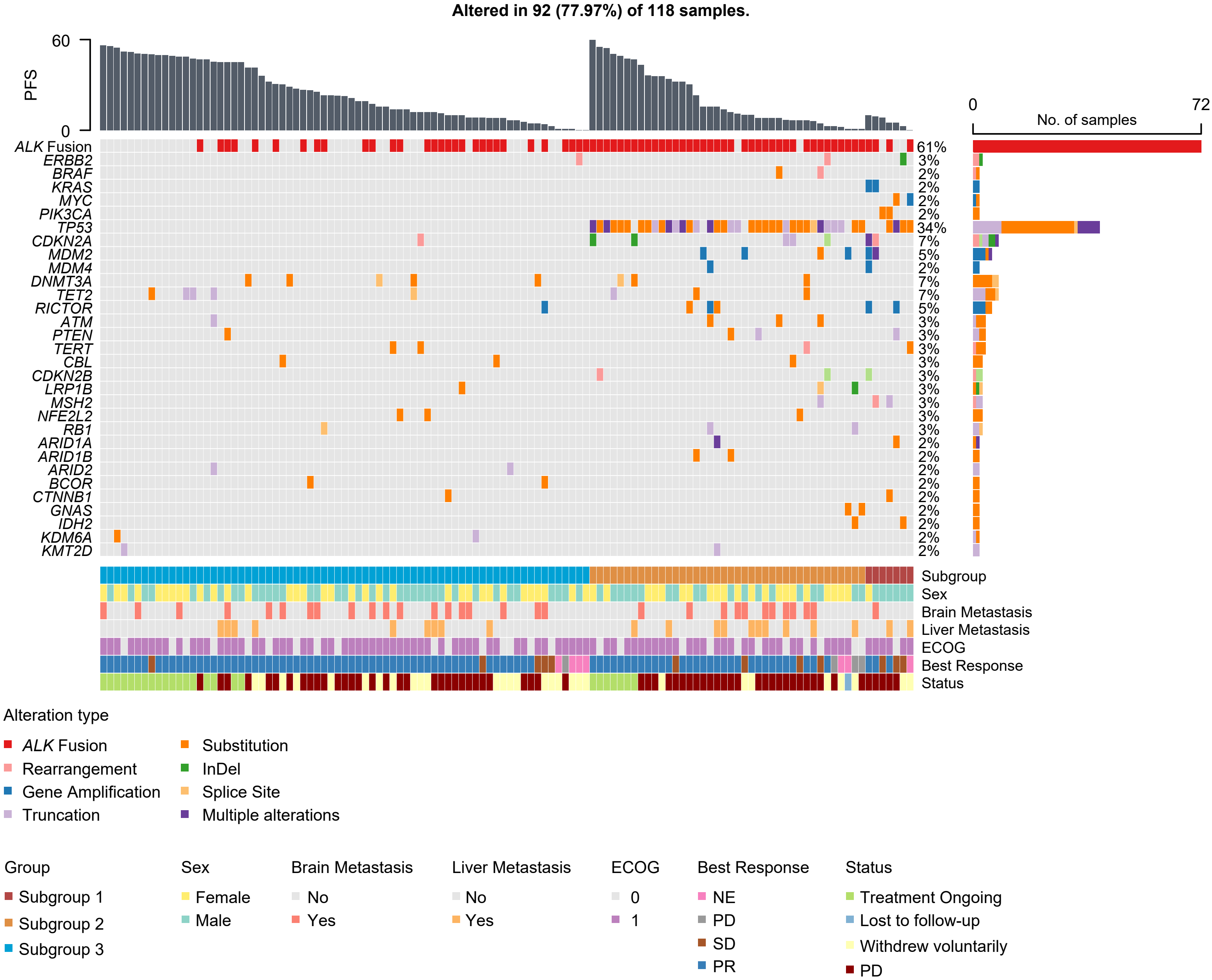

Figure S2. The mutation landscape at baseline in the envonalkib group.

Subgroup 1, patients with concurrent oncogenic driver alterations other than *ALK* fusions detected in ctDNA; Subgroup 2, patients with no concurrent oncogenic drivers detected in ctDNA, but with alterations resulting in p53 dysfunction; Subgroup 3, patients lacking both concurrent oncogenic driver and p53 pathway-related alterations detected in ctDNA.

Abbreviations: ALK, anaplastic lymphoma kinase; ECOG, Eastern Cooperative Oncology Group; PD, progressive disease; PR, partial response; SD, stable disease; NE, not evaluable.

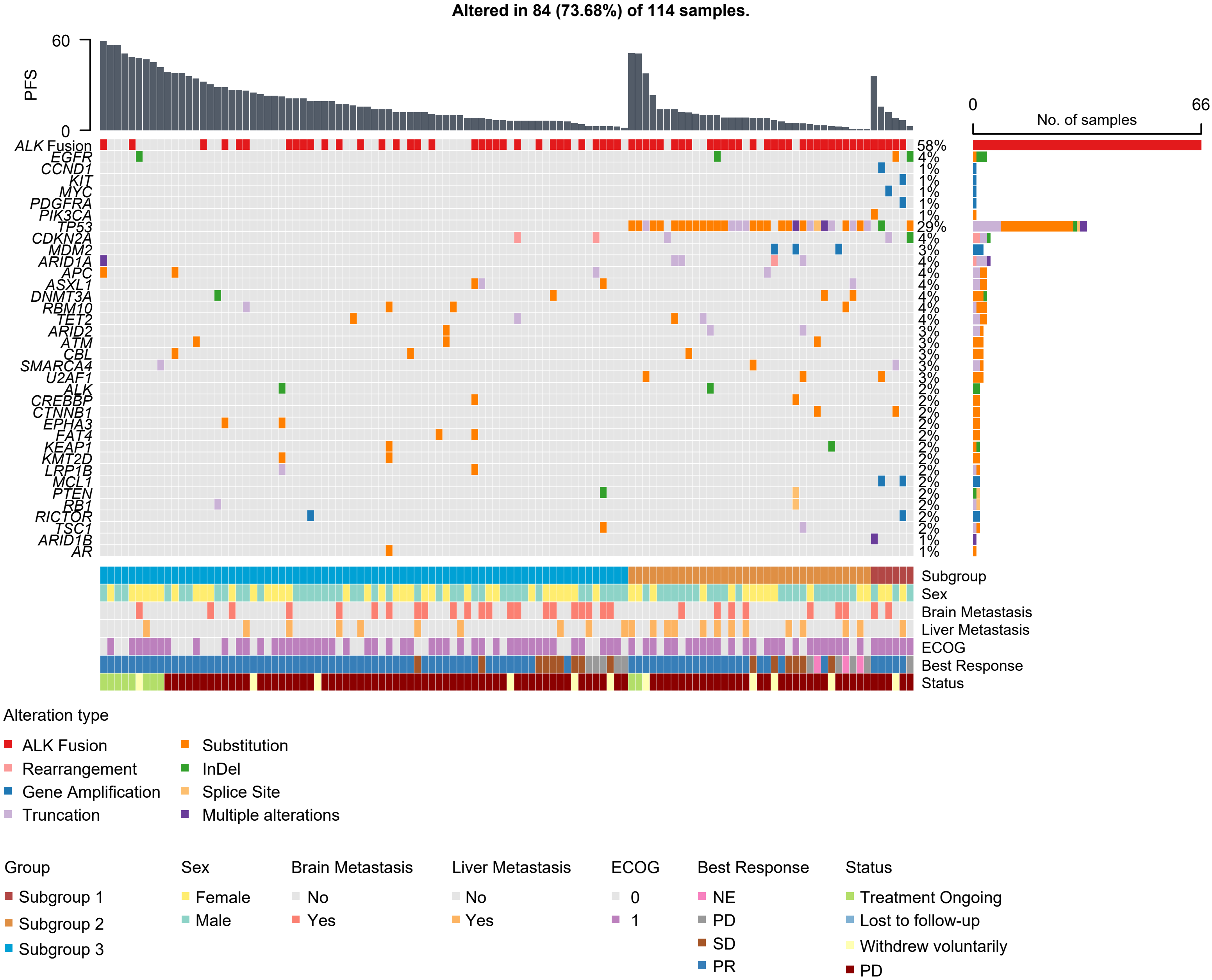

Figure S3. The mutation landscape at baseline in the crizotinib group.

Subgroup 1, patients with concurrent oncogenic driver alterations other than *ALK* fusions detected in ctDNA; Subgroup 2, patients with no concurrent oncogenic drivers detected in ctDNA, but with alterations resulting in p53 dysfunction; Subgroup 3, patients lacking both concurrent oncogenic driver and p53 pathway-related alterations detected in ctDNA. Abbreviations: ALK, anaplastic lymphoma kinase; ECOG, Eastern Cooperative Oncology Group; PD, progressive disease; PR, partial response; SD, stable disease; NE, not evaluable.

A

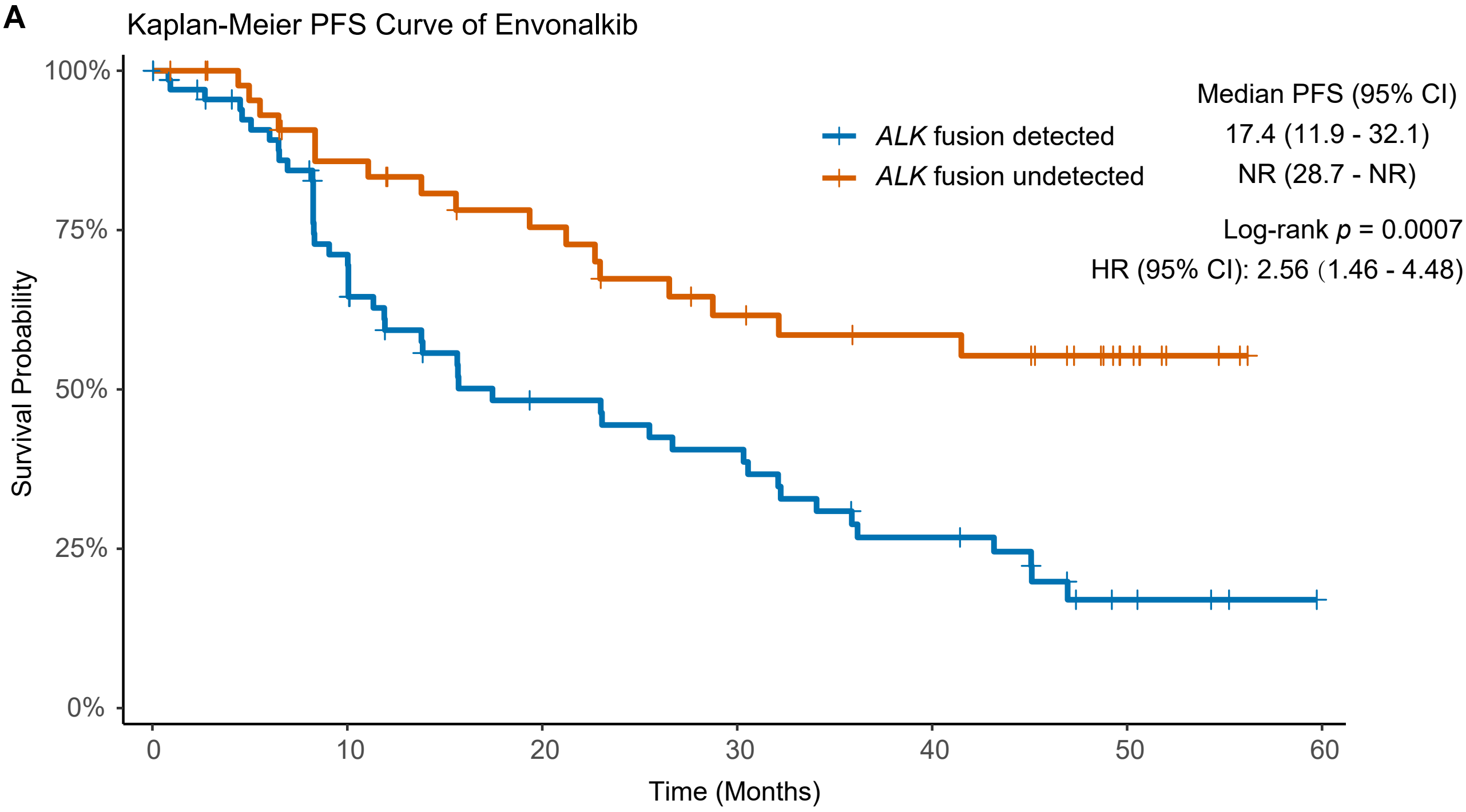

Number at Risk

	0	10	20	30	40	50	60
ALK fusion detected	72	43	25	21	13	4	0
ALK fusion undetected	46	35	28	21	18	8	0

B

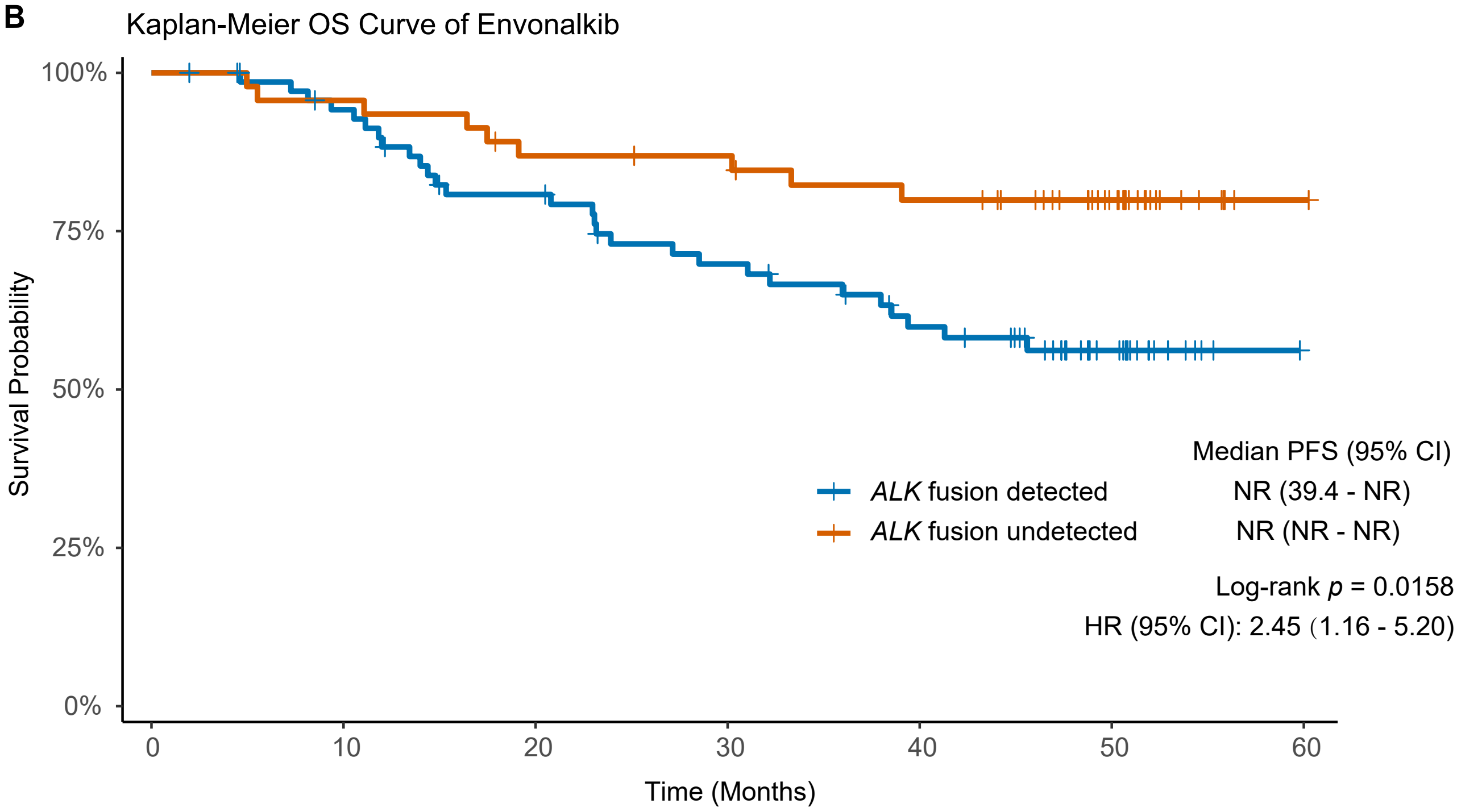

Number at Risk

	0	10	20	30	40	50	60
ALK fusion detected	72	64	53	44	35	16	0
ALK fusion undetected	46	44	39	38	34	21	1

Figure S4. Kaplan-Meier plots for patients with *ALK* fusions or without *ALK* fusions in the biomarker-evaluable population from envonalkib cohort.

(A) Kaplan-Meier curves of progression-free survival in the envonalkib group. (B) Kaplan-Meier curves of overall survival in the envonalkib group.
Abbreviations: PFS, progression-free survival; OS, overall survival; ALK, anaplastic lymphoma kinase; HR, hazard ratio; CI, confidence interval; NR, not reached.

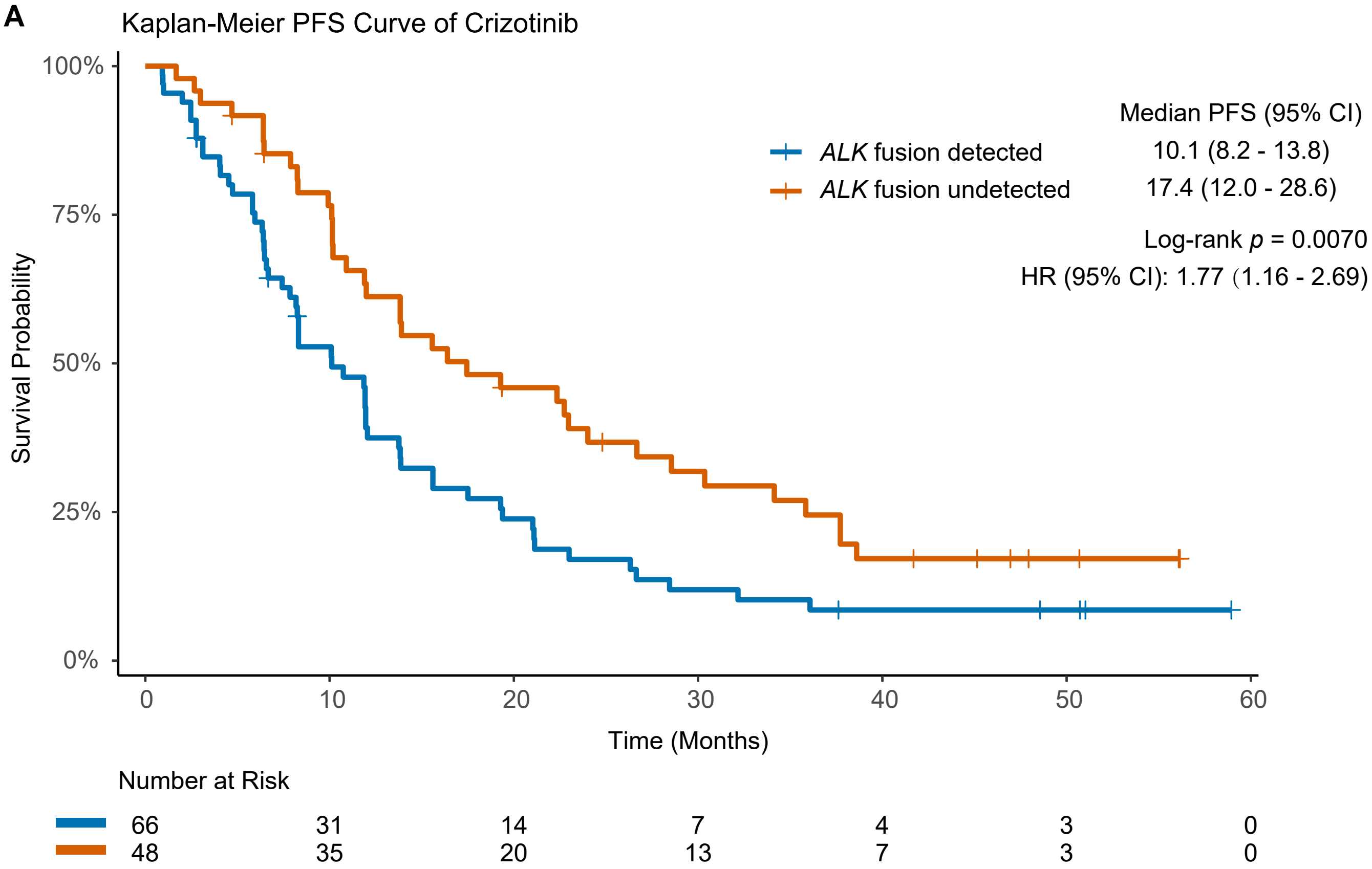

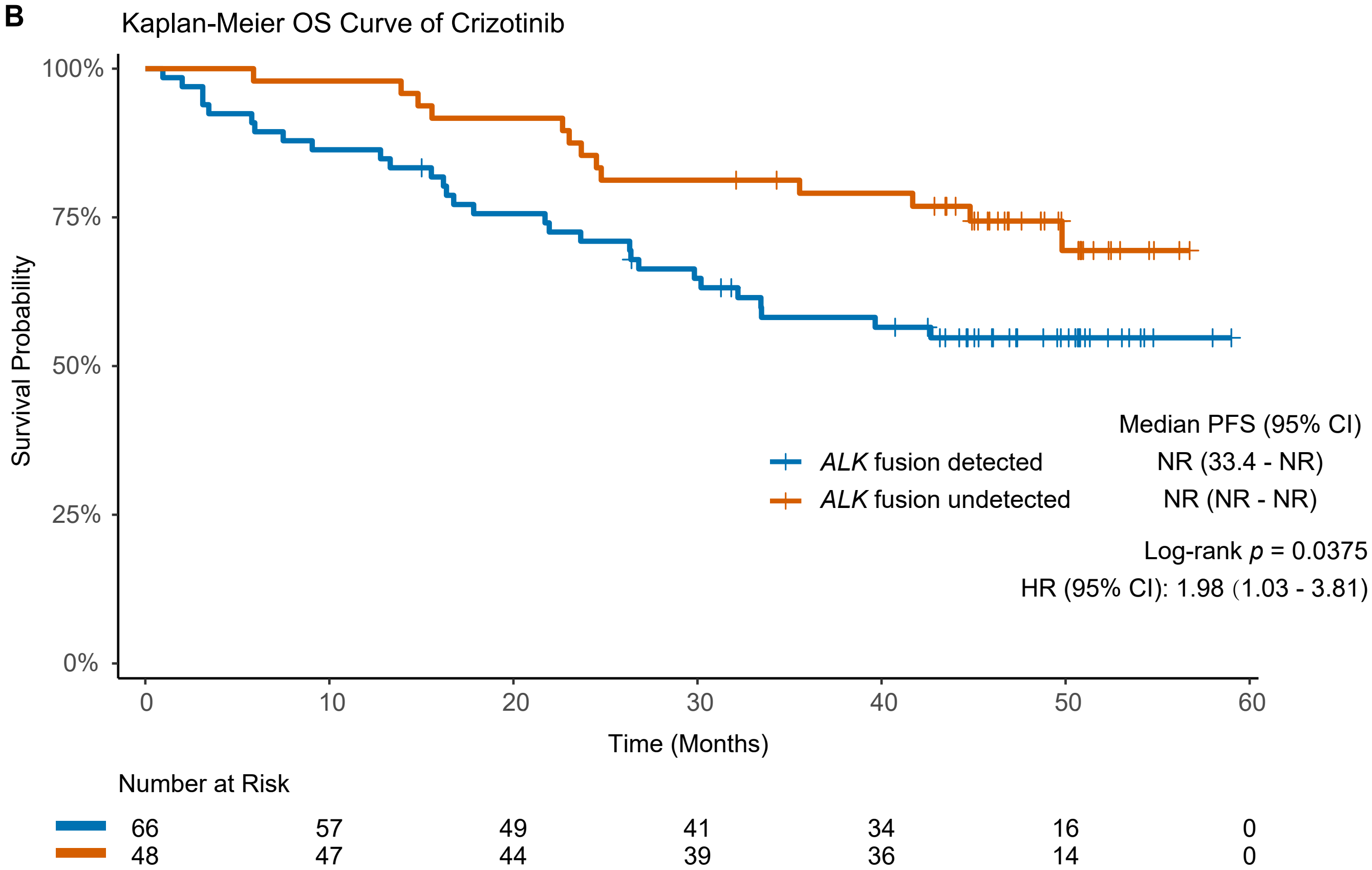

Figure S5. Kaplan-Meier plots for patients with *ALK* fusions or without *ALK* fusions in the biomarker-evaluable population from crizotinib cohort.

(A) Kaplan-Meier curves of progression-free survival in the crizotinib group. (B) Kaplan-Meier curves of overall survival in the crizotinib group.
Abbreviations: PFS, progression-free survival; OS, overall survival; ALK, anaplastic lymphoma kinase; HR, hazard ratio; CI, confidence interval; NR, not reached.

A Multivariable Cox PFS analysis of envonalkib

Characteristic	HR (95%CI)	*P* value
Age (years)	0.88 (0.68-1.13)	0.317
Sex		
Women	Reference	
Men	0.75 (0.40-1.40)	0.372
ECOG		
0	Reference	
1	0.69 (0.38-1.25)	0.217
Tumor burden (10mm)	1.07 (1.01-1.13)	0.027
Brain metastases		
No	Reference	
Yes	2.17 (1.25-3.76)	0.006
Previous chemotherapy		
No	Reference	
Yes	1.08 (0.59-2.00)	0.799
Smoking status		
Never	Reference	
Current/Former	1.56 (0.77-3.17)	0.217
ALK Fusions		
No	Reference	
Yes	2.28 (1.24-4.21)	0.008

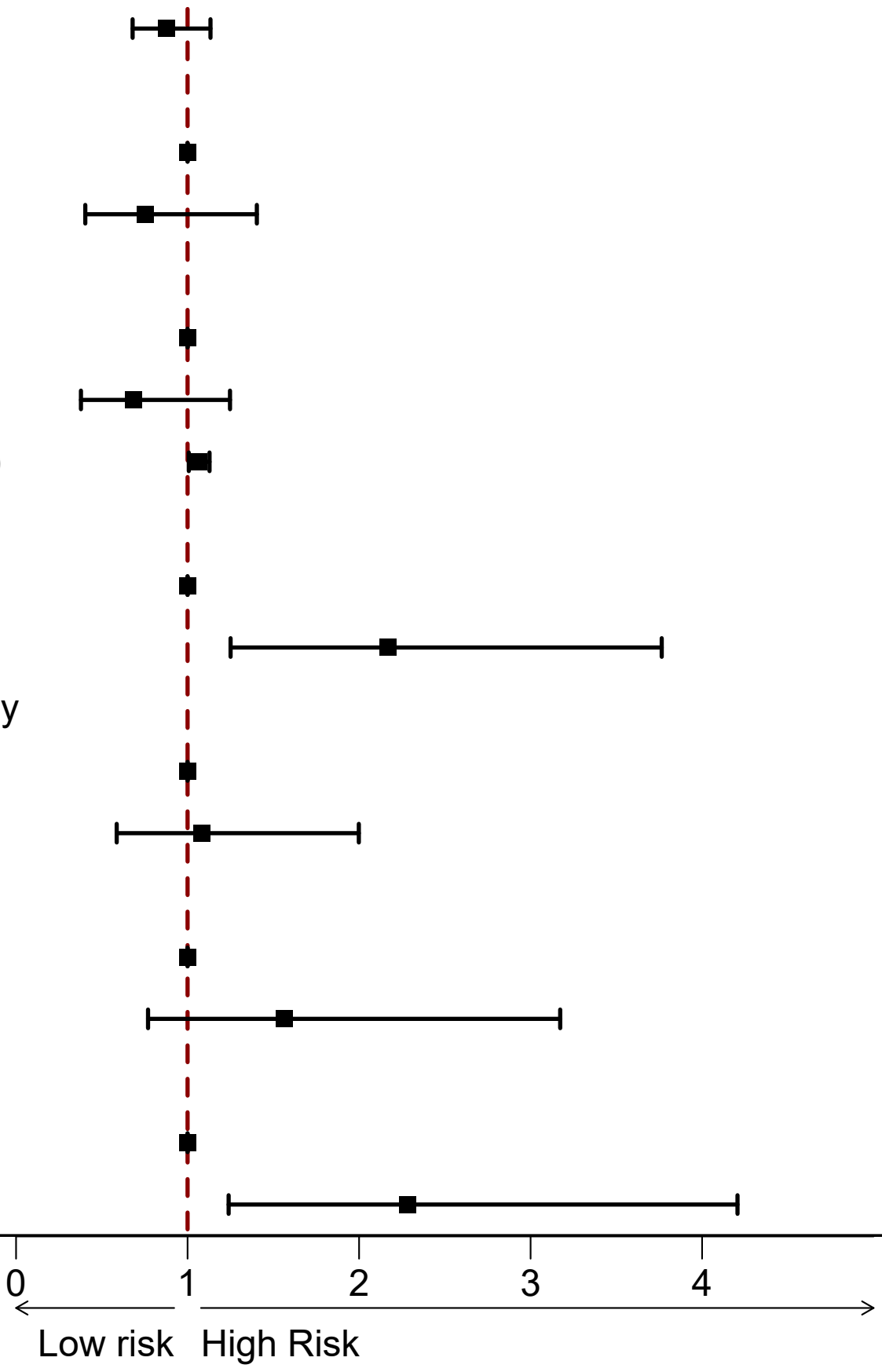

B Multivariable Cox PFS analysis of crizotinib

Characteristic	HR (95%CI)	*P* value
Age (years)	0.85 (0.69-1.03)	0.099
Sex		
Women	Reference	
Men	1.19 (0.59-2.39)	0.629
ECOG status		
0	Reference	
1	1.02 (0.66-1.59)	0.913
Tumor burden (10mm)	1.07 (1.00-1.13)	0.040
Brain metastases		
No	Reference	
Yes	1.61 (1.02-2.55)	0.040
Previous chemotherapy		
No	Reference	
Yes	1.36 (0.84-2.20)	0.206
Smoking status		
Never	Reference	
Current/Former	1.05 (0.52-2.10)	0.897
ALK Fusions		
No	Reference	
Yes	1.61 (1.03-2.51)	0.037

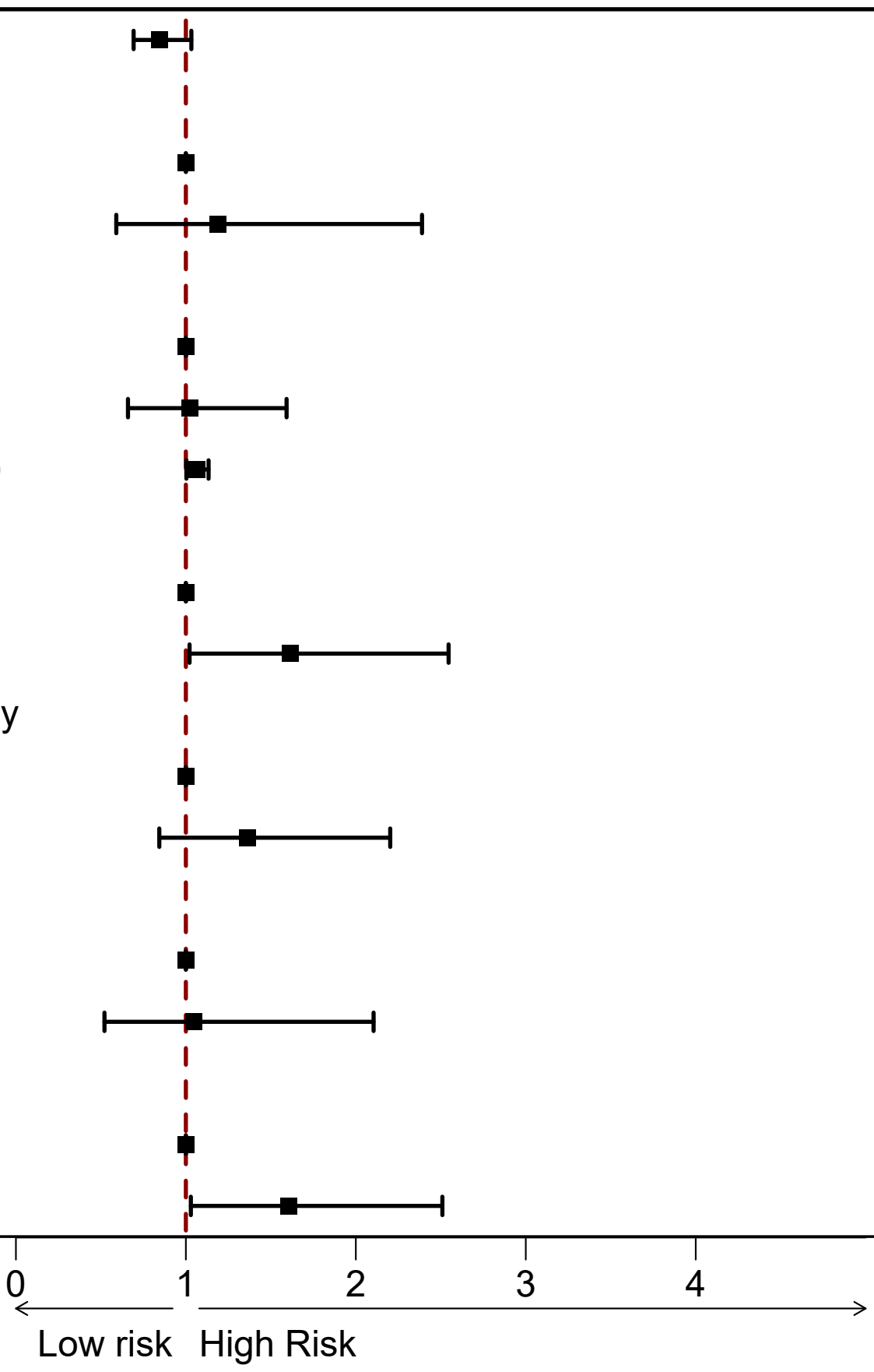

Figure S6. Multivariable Cox regression analysis of PFS by ctDNA *ALK* fusion status and clinical factors.

(A) Envonalkib arm. (B) Crizotinib arm.
Abbreviations: PFS, progression-free survival; ALK, anaplastic lymphoma kinase; HR, hazard ratio; CI, confidence interval; ECOG, Eastern Cooperative Oncology Group; ctDNA, circulating tumor DNA.

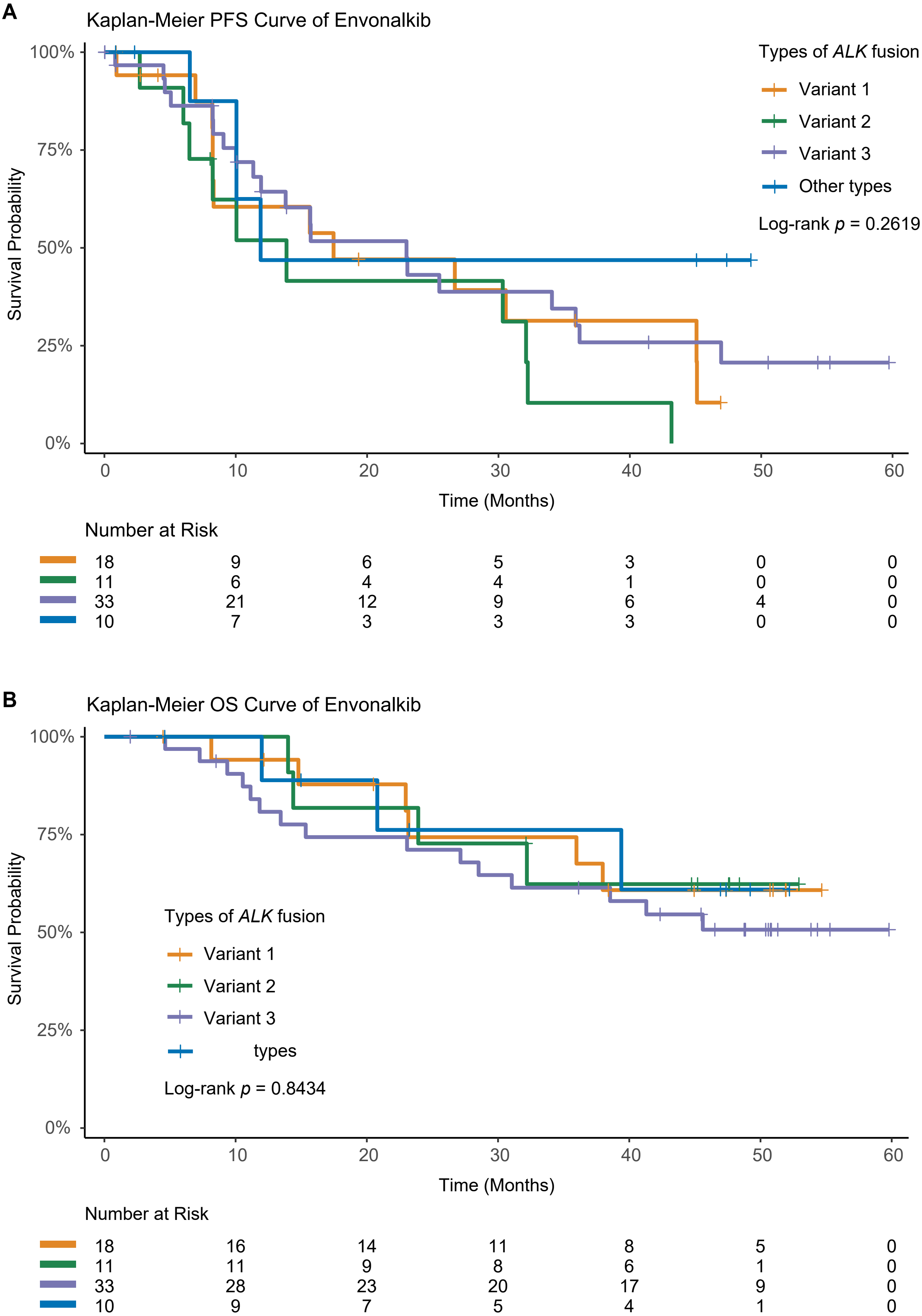

Figure S7. Kaplan-Meier plots for patients with different *ALK* variants in the biomarker-evaluable population from envonalkib cohort.

(A) Kaplan-Meier curves of progression-free survival in the envonalkib group. (B) Kaplan-Meier curves of overall survival in the envonalkib group.
Abbreviations: PFS, progression-free survival; OS, overall survival; ALK, anaplastic lymphoma kinase.

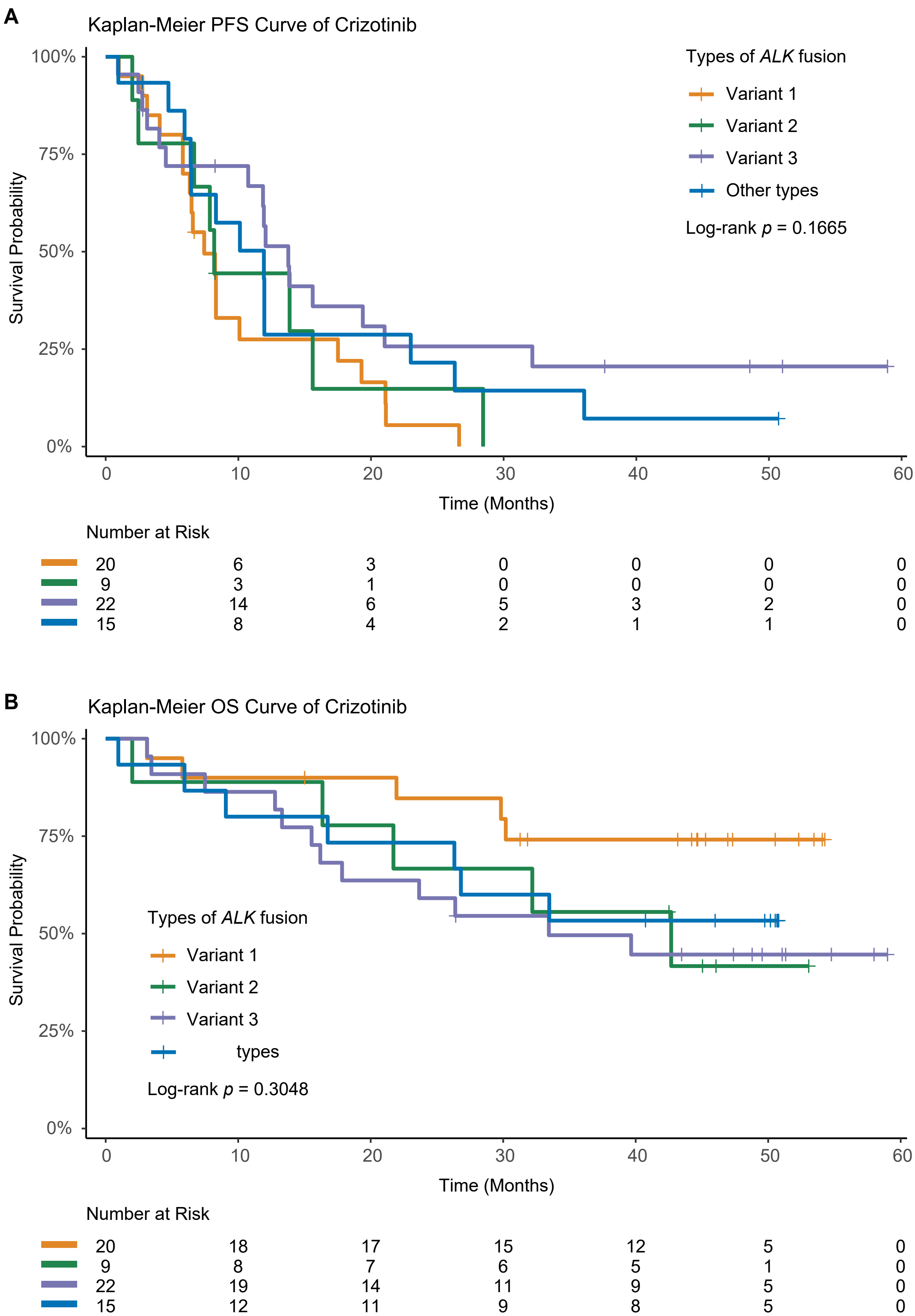

Figure S8. Kaplan-Meier plots for patients with different *ALK* variants in the biomarker-evaluable population from crizotinib cohort.

(A) Kaplan-Meier curves of progression-free survival in the crizotinib group. (B) Kaplan-Meier curves of overall survival in the crizotinib group.
Abbreviations: PFS, progression-free survival; OS, overall survival; ALK, anaplastic lymphoma kinase.

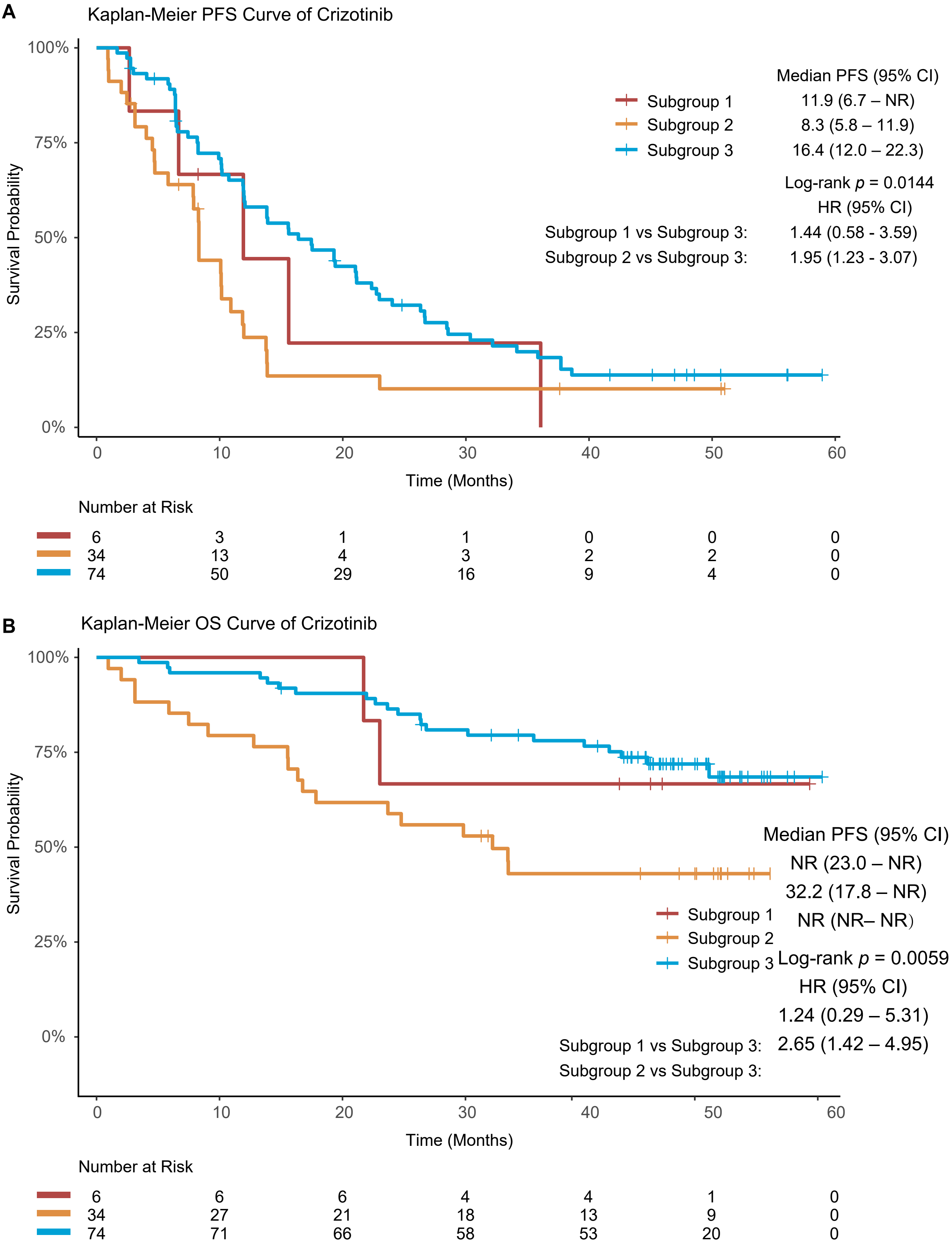

Figure S9. Kaplan-Meier plots for progression-free survival and overall survival in the biomarker-evaluable population from crizotinib cohort.

(A) Kaplan-Meier curves of progression-free survival. (B) Kaplan-Meier curves of overall survival. The sample was divided into three molecular subgroups: Subgroup 1, patients with concurrent oncogenic driver alterations other than *ALK* fusions detected in ctDNA; Subgroup 2, patients with no concurrent oncogenic drivers detected in ctDNA, but with alterations resulting in p53 dysfunction; Subgroup 3, patients lacking both concurrent oncogenic driver and p53 pathway-related alterations detected in ctDNA. Abbreviations: PFS, progression-free survival; OS, overall survival; ALK, anaplastic lymphoma kinase; HR, hazard ratio; CI, confidence interval; NR, not reached.

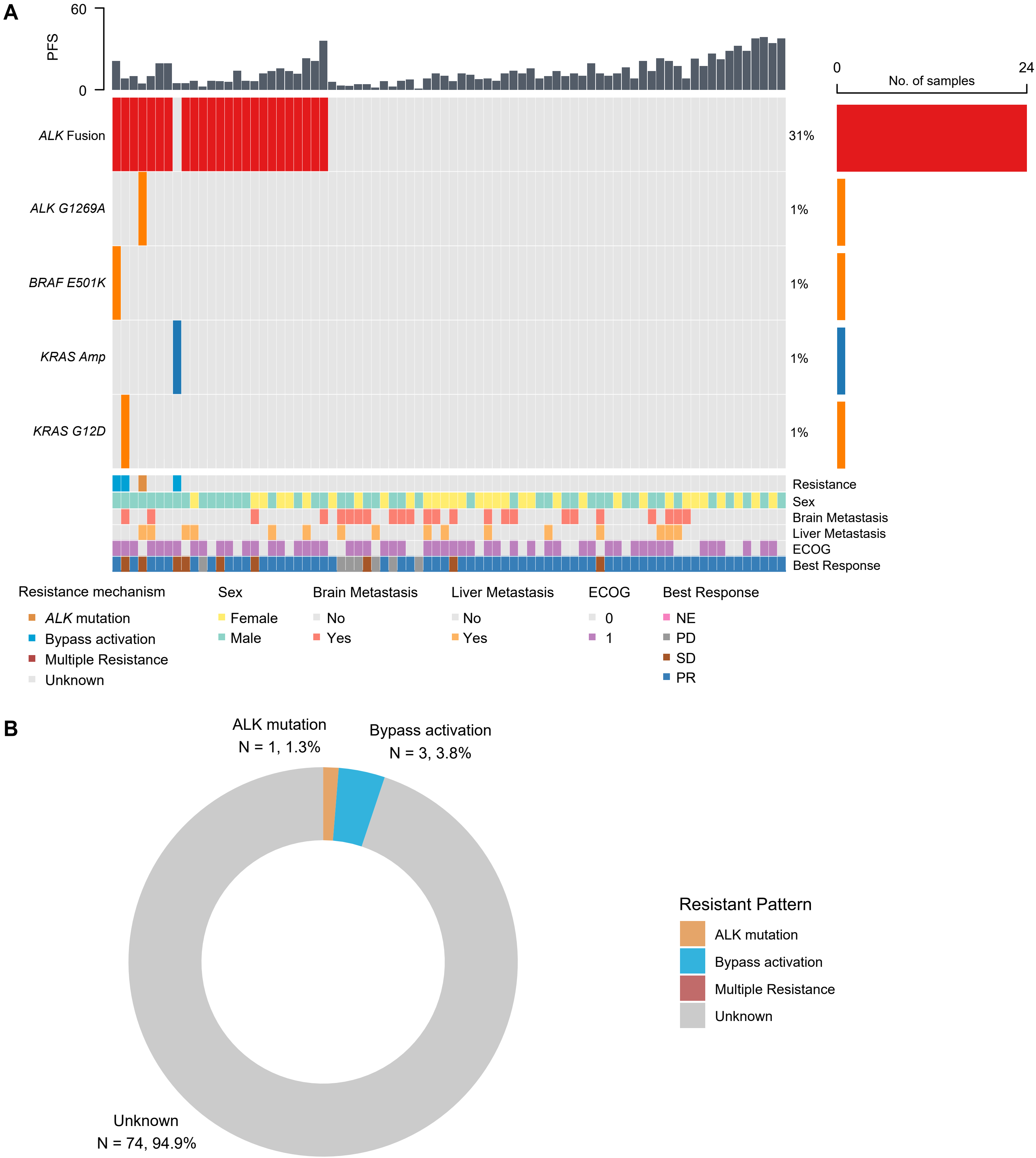

Figure S10. The mutational landscape at the time of disease progression in the crizotinib group.

Abbreviations: ALK, anaplastic lymphoma kinase; ECOG, Eastern Cooperative Oncology Group; PD, progressive disease; PR, partial response; SD, stable disease; NE, not evaluable.
